# Controlled Peptide-Mediated Vesicle Fusion Assessed by Simultaneous Dual-Colour Time-Lapsed Fluorescence Microscopy

**DOI:** 10.1038/s41598-020-59926-z

**Published:** 2020-02-20

**Authors:** Nestor Lopez Mora, Aimee L. Boyle, Bart Jan van Kolck, Anouk Rossen, Šárka Pokorná, Alena Koukalová, Radek Šachl, Martin Hof, Alexander Kros

**Affiliations:** 1 Supramolecular and Biomaterials Chemistry, Leiden Institute of Chemistry, Leiden University, P.O. Box 9502, 2300 RA Leiden, The Netherlands; 2 J. Heyrovský Institute of Physical Chemistry, Academy of Sciences of the Czech Republic, v.v.i., Dolejškova 2155/3, 182 23, Prague, 8 Czech Republic; 3 Macromolecular Biochemistry, Leiden Institute of Chemistry, Leiden University, P.O. Box 9502, 2300 RA Leiden, The Netherlands

**Keywords:** Membrane biophysics, Membranes

## Abstract

We have employed a model system, inspired by SNARE proteins, to facilitate membrane fusion between Giant Unilamellar Vesicles (GUVs) and Large Unilamellar Vesicles (LUVs) under physiological conditions. In this system, two synthetic lipopeptide constructs comprising the coiled-coil heterodimer-forming peptides K_4_, (KIAALKE)_4_, or E_4_, (EIAALEK)_4_, a PEG spacer of variable length, and a cholesterol moiety to anchor the peptides into the liposome membrane replace the natural SNARE proteins. GUVs are functionalized with one of the lipopeptide constructs and the fusion process is triggered by adding LUVs bearing the complementary lipopeptide. Dual-colour time lapse fluorescence microscopy was used to visualize lipid- and content-mixing. Using conventional confocal microscopy, lipid mixing was observed on the lipid bilayer of individual GUVs. In addition to lipid-mixing, content-mixing assays showed a low efficiency due to clustering of K_4_-functionalized LUVs on the GUVs target membranes. We showed that, through the use of the non-ionic surfactant Tween 20, content-mixing between GUVs and LUVs could be improved, meaning this system has the potential to be employed for drug delivery in biological systems.

## Introduction

Numerous model systems for membrane fusion have been developed in recent years, all of which have employed a diverse range of molecules as fusogens. Examples of such fusogens include: hydrogen bonding motifs^1^, DNA^2–11^, PNA^12–14^, coiled-coil peptides^15–18^, and small molecule recognition motifs^19–23^. These systems exhibit varying efficiencies of fusion: some only facilitate hemifusion, or lipid-mixing; whereas others promote full fusion, resulting in content-mixing. For systems that demonstrate full fusion, not all are able to do so specifically, *i.e*. content-mixing is often accompanied by leakage.

**Figure 1:**
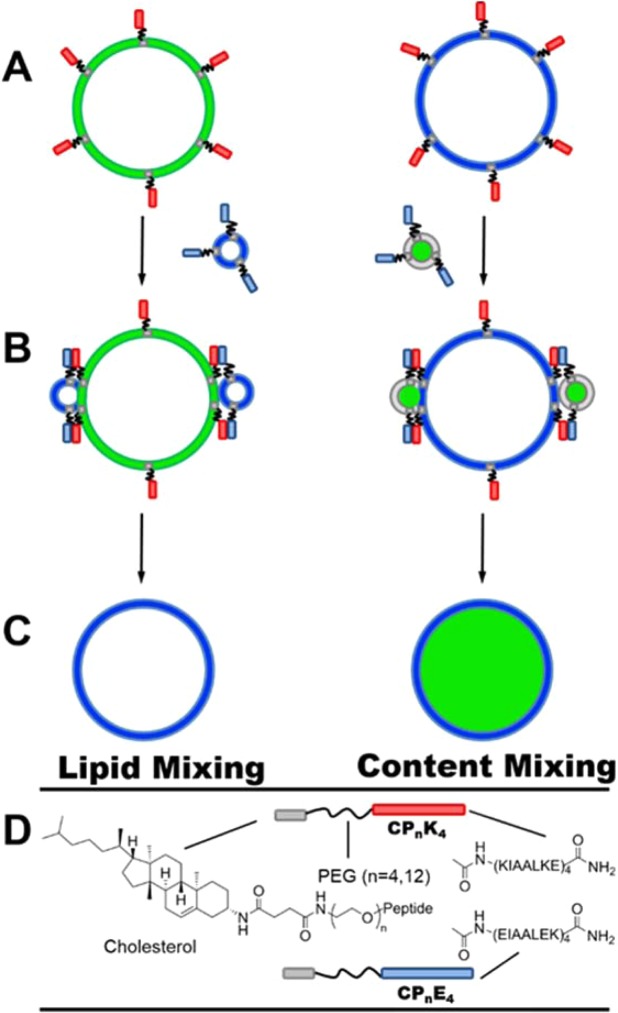
Schematic representation of coiled-coil peptide-mediated membrane fusion between GUVs and LUVs. GUVs are functionalized with lipopeptide CP_n_K_4_ (red) and LUVs with CP_n_E_4_ (blue). Lipid-mixing is shown on the left and content-mixing on the right of the upper panel. (**A**) Spontaneous incorporation of the lipidated peptide into the lipid membrane via the cholesterol anchor results in the functionalization of GUVs with CP_n_K_4_. (**B**) Addition of CP_n_E_4_-functionalized LUVs to CP_n_K_4_-functionalized GUVs leads to the formation of a coiled-coil complex which triggers fusion. (**C**) Transfer of the fluorescent lipids and mixing of the inner aqueous contents of the GUV after fusion with LUVs represents lipid- and content-mixing. (**D**) The structures of the CP_n_K_4_ and CP_n_E_4_ lipopeptides employed in this study.

We have previously developed a model system capable of specific, leakage-free, full fusion, which was inspired by SNARE-driven membrane fusion. This system comprises two coiled-coil-forming peptides named K_4_ (KIAALKE)_4_ and E_4_ (EIAALEK)_4_^24^, which serve as fusogenic recognition motifs. These are coupled to a cholesterol membrane anchor via a flexible polyethylene glycol (PEG) linker of variable length (Fig. 1), to yield the constructs CP_n_K_4_ and CP_n_E_4_. The formation of a heterodimeric coiled-coil brings the two opposing membranes into close proximity, inducing efficient, leakage-free, membrane fusion^25^. Numerous aspects of this system, including: the length and oligomer state of the peptides^26,27^, the PEG spacer length^28^, the nature and position of the lipid anchor^29–31^, and the effects of these variables on the fusion process have been investigated. Moreover, the system has been employed for the targeted delivery of various cargoes to the membranes of GUVs and cells^32–35^.

To date, Large Unilamellar Vesicles (LUVs), with sizes of approximately 100 nm, have been used to probe the various aspects of this model system^36–39^; however, the LUV–LUV interaction exhibits an inherent high degree of membrane curvature and tension, which may affect the energetics of the membrane fusion process. By employing Giant Unilamellar Vesicles (GUVs) and mixing them with LUVs, fusion could be visualized in more detail at the lipid bilayer, and would also be more relevant to natural fusion processes as the size of a typical GUV ranges from 1–100 µm, which is more representative of the size of a cell (1–10 µm). There are a limited number of fusion studies which have been conducted with GUVs and LUVs, employing either natural SNARE proteins^40–43^, or amphipathic, monomeric peptides based on viral fusion protein sequences^44,45^. To the best of our knowledge, no designed, multi-component peptide system has been used to induce fusion between GUVs and LUVs. In addition, the use of DexPEG hydrogels allows the growth of GUVs in good yields under high ionic strength conditions^46^, which are required for lipid- and content-mixing assays.

Herein, we use conventional confocal microscopy to monitor membrane fusion of GUVs and LUVs in time-lapse fluorescence microscopy experiments at physiologically relevant ionic strengths, using CaCl_2_ and MgCl_2_ supplemented PBS. The peptide-functionalized GUVs (sizes 5–20 µm), act as a simple biophysical model of the plasma membrane of cells, and the fusion process is triggered upon the addition of LUVs bearing the complementary lipopeptide. Membrane fusion, promoted by our synthetic coiled-coil peptide system, was tested *via* lipid-mixing assays, and the mixing of the inner aqueous contents through content-mixing assays by utilizing simultaneous dual-color fluorescence microscopy experiments (Fig. 1).

## Results and Discussion

### Lipid-mixing assays show GUVs and LUVs exchange lipids

**Figure 2:**
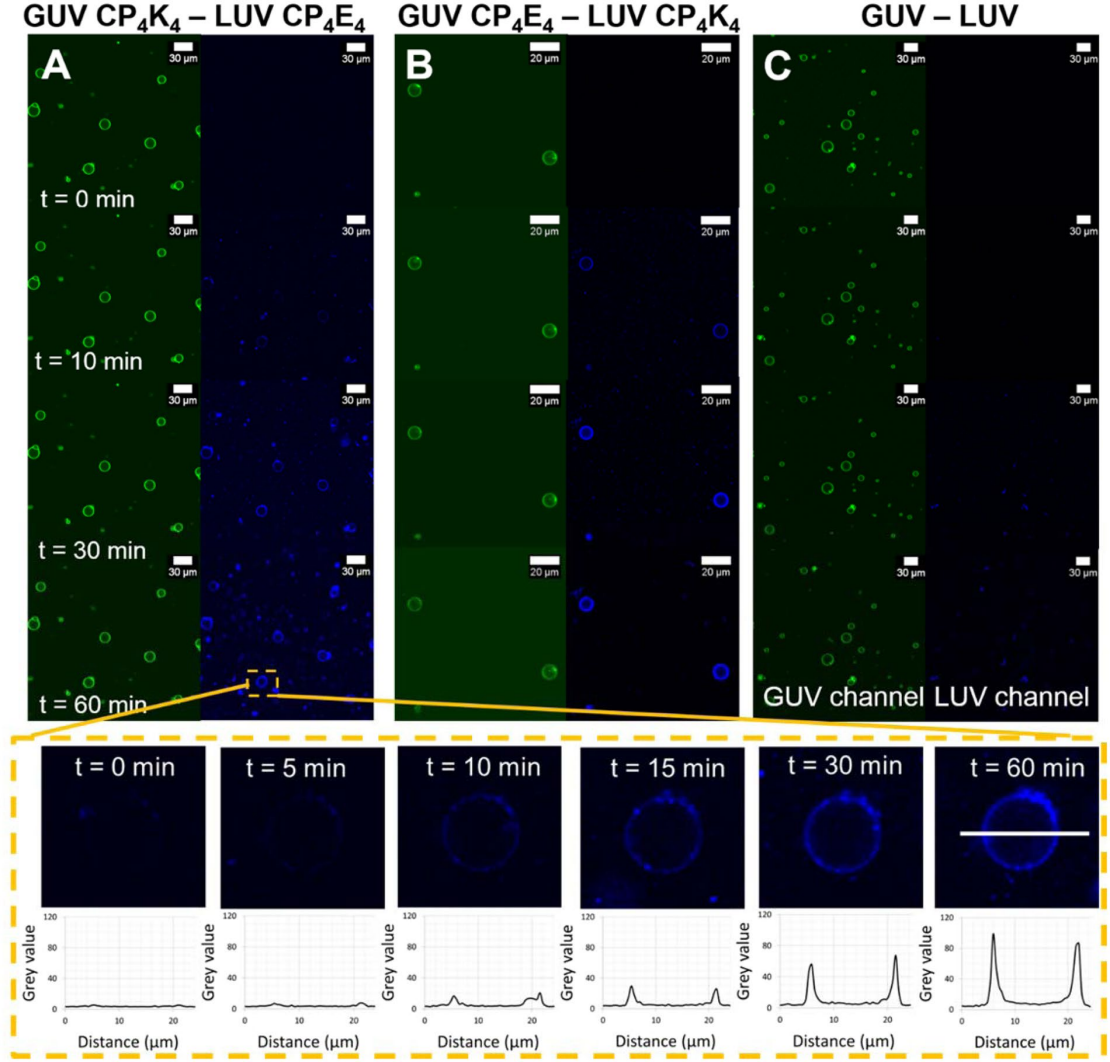
Time-lapse micrographs of the lipid-mixing assay between lipopeptide-functionalized GUVs and lipopeptide-functionalized LUVs before (time = 0 minutes) and after (time = 10, 30 and 60 minutes) appearance of LUVs in the confocal volume. The GUVs are excited at 488 nm and fluorescence emission is detected between 500–550 nm (green), while LUVs are excited at 633 nm and emission is detected between 650–700 nm (blue). The lower panels show a selected GUV magnified at time = 0, 5, 10, 15, 30 and 60 minutes during the time-lapse lipid-mixing experiment and the corresponding fluorescence intensity evolution along the cross-section of the lipid bilayer is plotted in the graphs. (**A**) Lipid-mixing assay between CP_4_K_4_-functionalized GUVs and CP_4_E_4_-functionalized LUVs, (**B**) lipid-mixing assay between CP_4_E_4_-functionalized GUVs and CP_4_K_4_-functionalized LUVs, (**C**) lipid-mixing assay between non-functionalized GUVs and non-functionalized LUVs. Imaging was performed every minute for one hour using a Leica TCS SPE microscope.

Lipid mixing was detected simultaneously by dual-color fluorescence imaging of peptide-functionalized GUVs incorporating ATTO 488 DOPE and peptide-functionalized LUVs labeled with ATTO 633 DOPE. Initially, the appearance of ‘spotty patches’ and non-homogeneously distributed faint fluorescence on the lipid bilayer of the GUVs indirectly suggests liposome docking, which is not directly detectable due to the resolution limit of optical microscopy. After 30 minutes, the majority of the GUVs showed a sharp fluorescence increase, homogeneously distributed over the entire lipid bilayer, and this fluorescence intensity increased after 60 minutes indicating lipid-mixing was occurring (Fig. 2A and GUV magnification). The presence of intact GUVs was verified by observing fluorescence in the green channel at the end of the assay. The size of individual GUVs was measured directly from the micrographs before and after the lipid-mixing assay, and no size difference in the diameter of the GUVs was detected. This is due to the large size of the GUVs (10 µm), which is two orders of magnitude larger than that of the liposomes (100 nm), with GUV volume scaling with the third power of the radius. Interchanging the lipopeptides, *i.e*. functionalizing GUVs with CP_4_E_4_ and LUVs with CP_4_K_4_, also resulted in lipid mixing (Fig. 2B). Switching the lipopeptides in this manner resulted in the earlier appearance of spots and full lipid mixing after just 10 minutes, suggesting that CP_4_K_4_-functionalized LUVs interact more strongly with GUVs containing the complementary lipopeptide in comparison to CP_4_E_4_-functionalized LUVs. This indicates asymmetric roles of the lipopeptides during lipid mixing assay. This result correlates well with previous studies which uncovered distinct but complementary roles of the lipopeptides during initial steps of membrane fusion: E_4_ is exposed to the bulk and solely promotes membrane binding of CP_n_K_4_; while K_4_ loops back to the lipid–water interface where it promotes bilayer contact by binding to CP_n_E_4_ containing bilayers and initiates membrane fusion by modulating the bilayer properties^47^.

Similar experimental conditions were used for a control experiment where non-functionalized GUVs and LUVs were mixed (Fig. 2C). Some non-homogeneous fluorescence was observed in the target GUV lipid bilayer after one hour indicating a non-specific interaction, likely promoted by negative membrane curvature due to the presence of DOPE in both GUVs and LUVs. As an additional control experiment, the lipopeptide was omitted from the target membrane of the GUVs and these were mixed with CP_4_E_4_-functionalized LUVs. Fluorescence imaging showed that these CP_4_E_4_-functionalized LUVs have minimal interaction with the non-functionalized GUV membrane, Supplementary Fig. S1. Moreover, we performed the converse lipid-mixing assay by mixing CP_4_K_4_-functionalized GUVs with non-functionalized LUVs (Supplementary Fig. S1). The fluorescence imaging showed that plain LUVs interact strongly with the CP_4_K_4_-functionalized GUVs, transferring the fluorescent lipid (ATTO 633 DOPE) to the membrane of the GUVs after 30 minutes. These control experiments reveal that K_4_ has a strong interaction with the membrane of GUVs, resulting in non-specific interactions with LUVs. This interaction has been previously reported in lipid monolayer studies combined with surface sensitive infrared reflection absorption spectroscopy (IRRAS) and liposomal assays^48,49^. Therefore, for this system to be highly specific, it is important that the target membrane is functionalized with CP_4_E_4_ and LUVs contain CP_4_K_4_.

### Content mixing is promoted by the addition of Tween 20

**Figure 3:**
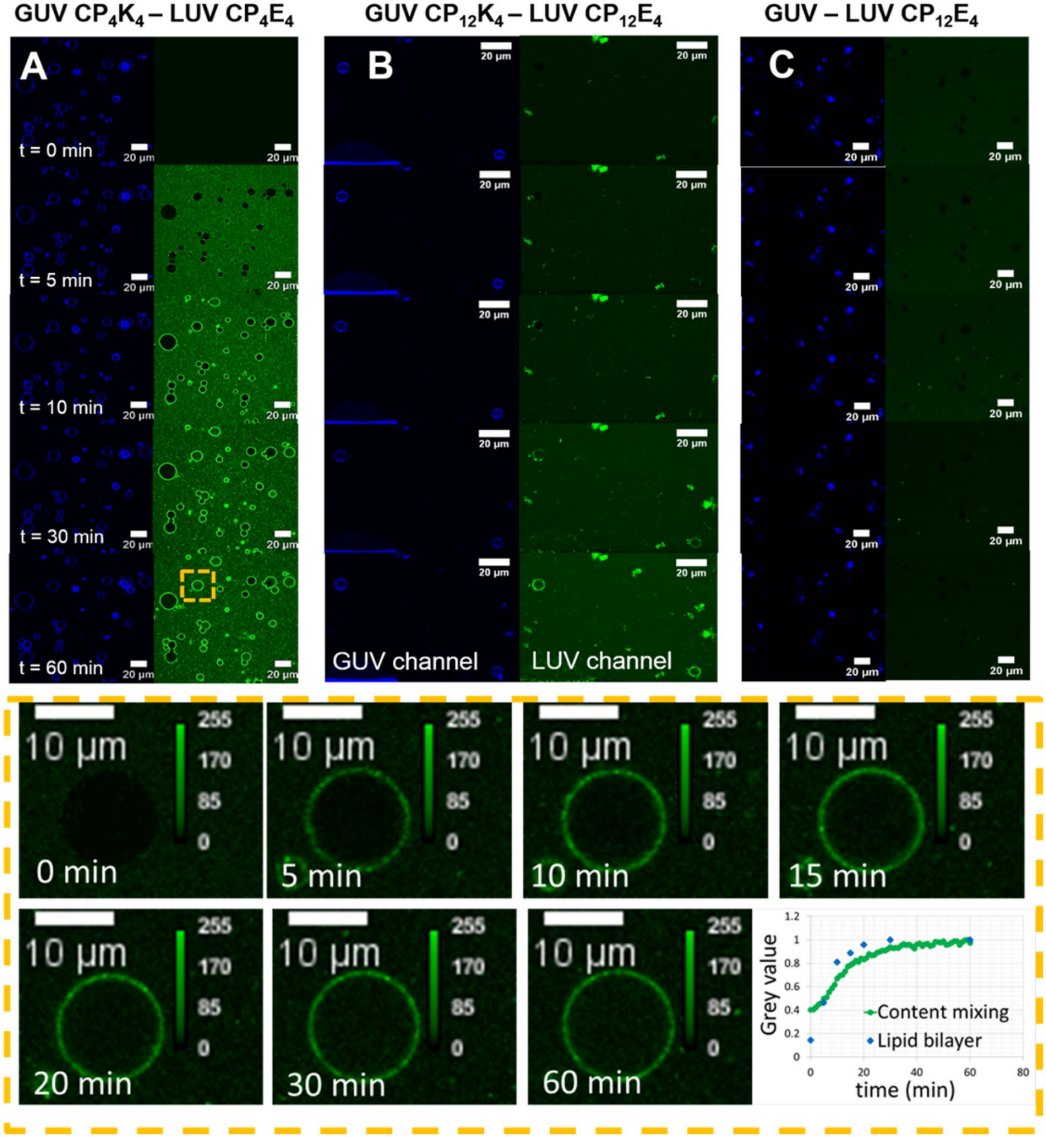
Time-lapse micrographs of the content-mixing assay between lipopeptide-functionalized GUVs and lipopeptide-functionalized LUVs before (time = 0) and after (time = 5, 10, 30 and 60 minutes) appearance of LUVs in the confocal volume. GUVs are excited at 633 nm and fluorescence emission is detected between 650–700 nm (blue), while LUVs are excited at 488 nm and the emission is detected between 500–550 nm (green). The lower panels show one GUV magnified at time = 0, 5, 10, 15, 20, 30 and 60 minutes during the time-lapse content-mixing experiment and a normalized plot of the fluorescence evolution in the lumen and lipid bilayer of the GUV has been constructed from these images (lower right panel). (**A**) Content mixing assay between CP_4_K_4_-functionalized GUVs and CP_4_E_4_-functionalized LUVs, (**B**) content-mixing assay between CP_12_K_4_-functionalized GUVs and CP_12_E_4_-functionalized LUVs, (**C**) control content-mixing assay between non-functionalized GUVs and CP_12_E_4_-functionalized LUVs.

In addition to lipid-mixing, this system was also evaluated for its ability to promote content mixing, which has been described as the ‘hallmark’ of true fusion and the K_4_ – membrane interaction was modulated with the increase of the length of the PEG spacer. The content-mixing experiment between CP_4_K_4_-functionalized GUVs and CP_4_E_4_-functionalized LUVs is presented in Fig. 3A. Because of the encapsulation of a high concentration of carboxyfluorescein inside the LUVs, a considerable increase in background fluorescence was detected immediately after the addition of carboxyfluorescein-loaded LUVs. This background fluorescence is likely to be due to free carboxyfluorescein being released from the permeable membrane of the LUVs, which is not efficiently removed after purification by size exclusion, and the interaction of peptide functionalized LUVs with the protein-passivated surface in the microscopy chamber. Fluorescence colocalization of LUVs in the target membrane of GUVs could be observed after 10 minutes, despite the LUVs not being labeled with a fluorescent lipid. This suggests that, after the merging of LUVs with GUVs, a fraction of the carboxyfluorescein from the LUVs is distributed along the lipid bilayer, depicting the boundaries of the GUV, (Fig. 3A, green channel t = 10 min). The timeframe of LUV fluorescence colocalization in the lipid bilayer of GUVs (GUV magnification in Fig. 3) correlates with the timeframe of the lipid-mixing assay (plot profiles in Fig. 2). Inner content mixing was observed after 30 minutes in the GUVs with small sizes (5–10 µm) and after 60 minutes in larger GUVs (>15 µm); inner content mixing was not observed in all the GUVs however. The time difference in the observation of full fusion between small and large GUVs can be attributed to their volume differences. A 10-fold increase in GUV diameter corresponds to a 1000-fold increase in the volume of a GUV^50^, so many more fusion events between LUVs and GUVs would need to occur in the 15 µm GUV, which is 27 times larger than a 5 µm GUV, in order to detect inner content mixing. In addition not all the vesicles are identical, these differences are averaged out in bulk measurements but in single-vesicle experiments such as this, differences in lipid composition^51,52^, and encapsulation efficiency^53,54^, for example can lead to large differences in the observed rate of fusion and even in the ability of a vesicle to fuse at all. The exclusion of lipopeptide CP_n_K_4_ from the membrane of the GUVs in control experiments did not lead to content-mixing, (Fig. 3C), which indicates that the presence of CP_n_K_4_ is essential for full fusion to occur.

**Figure 4:**
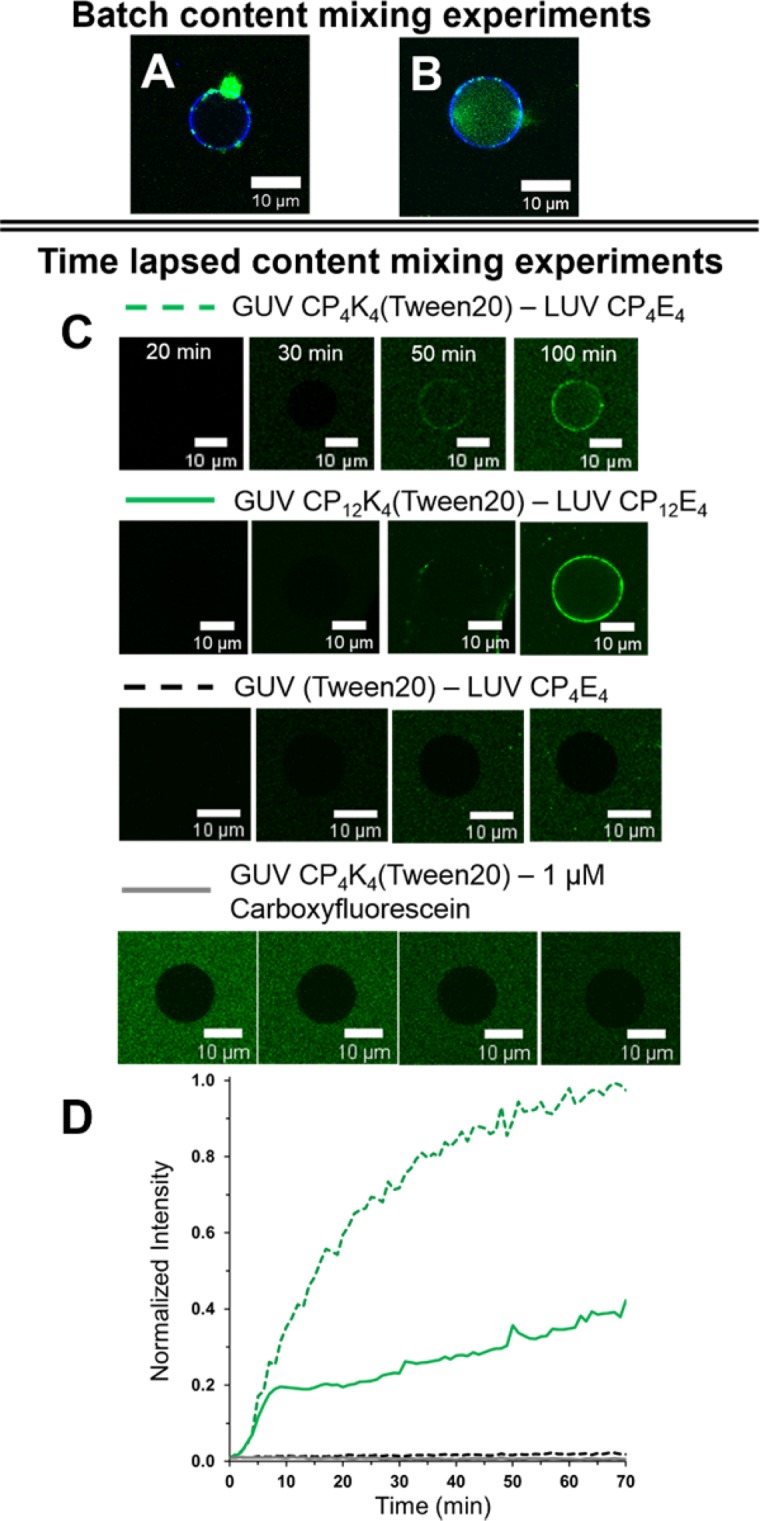
Batch and time-lapse content-mixing experiments. (**A**) Batch content-mixing assay after one hour of incubation of CP_4_K_4_-functionalized GUVs and CP_4_E_4_-functionalized LUVs. The micrograph is the overlay of fluorescence confocal microscopy images for 488 nm (green) and 633 nm (blue) channels that shows a single GUV in blue without content mixing and clustering of liposomes in green. (**B**) Content-mixing assay after one hour of incubation of CP_4_K_4_-Tween 20 functionalized GUVs and CP_4_E_4_-functionalized LUVs. The micrograph shows a single GUV in blue with content mixing in green in the lumen of the GUV. Less clustering of LUVs is observed when Tween 20 is present. (**C**) Time-lapsed fluorescence microscopy images of selected single GUV undergoing content mixing with: GUV CP_4_K_4_-Tween 20 – LUV CP_4_E_4,_ green dotted line; CP_12_K_4_-Tween 20 – LUV CP_12_E_4_, green solid line; GUV (Tween 20) – LUV CP_4_E_4_, black dotted line and GUV CP_4_K_4_-Tween 20 – 1 µM carboxyfluorescein, grey line. (**D**) Normalized fluorescence intensities over time in the lumen of single GUV from (**C**) Normalization profiles were calculated with the maximum fluorescence value obtained from the CP_4_K_4_-Tween 20 – LUV CP_4_E_4_ assay.

In an attempt to increase the quantity of GUVs undergoing content mixing, the length of the PEG spacer was changed from PEG_4_ to PEG_12_. The K_4_–membrane interaction is expected to decrease as a function of spacer length, positively impacting the membrane fusion efficiency as previously found for LUV-LUV fusion events^28^. Figure 3B shows the content mixing experiment between CP_12_K_4_-GUVs and CP_12_E_4_-functionalized LUVs loaded with carboxyfluorescein. Content mixing was observed in some vesicles with small sizes (5–10 µm) after 60 minutes, but this content mixing was not detected for all imaged GUVs. Analysis of individual GUVs after membrane fusion showed that GUVs that do not undergo content-mixing exhibit large amounts of clustered LUVs in the target membrane. In previous studies with batch content-mixing experiments, it was discovered that mixing CP_4_K_4_ and the detergent Tween 20 increases the fusion efficiency of the fusogenic system, probably by softening the GUV lipid bilayer^55^, and breaking up lipopeptide aggregates^56^ that are formed in the target membrane (Fig. 4A,B). We further studied this effect by preparing a CP_n_K_4_-Tween 20 (n = 4, 12) mixture with two different Tween 20 concentrations (0.4 and 1 mol% with respect to CP_n_K_4_) and incubated this mixture with GUVs. We then followed the same experimental procedure for the time-lapse content-mixing assay as employed previously. A better performance with 1 mol%, in comparison to 0.4 mol%, Tween 20 was observed (Supplementary Figs. S4–S7), supporting our hypothesis that membrane fusion efficiency can be improved with the incorporation of Tween 20. Time lapse fluorescence microscopy images of selected CP_4_K_4_-Tween 20-labelled GUVs (GUV 9, Supplementary Fig. S7) and CP_12_K_4_-Tween 20-labelled GUVs (GUV 1, Supplementary Video) undergoing content-mixing are presented in Fig. 4C. No LUV clustering was observed in these GUVs after 20 minutes, whereas in the absence of Tween 20 this clustering is detected after 10 minutes (see Fig. 3A,B). The fluorescence intensity in the lumen of the GUV increased after 25 minutes and plateaued after 60 minutes (Fig. 4D). Most of the small GUVs exhibited content mixing after 60 minutes of imaging while larger vesicles gave less, or no, fluorescence in agreement with the previous content-mixing experiments.

The effect on the target membrane of the GUVs of adding Tween 20 alone was evaluated by incubating plain GUVs with Tween 20 (1 mol% with respect to CP_n_K_4_) and mixing with carboxyfluorescein-loaded CP_4_E_4_-functionalized LUVs (Fig. 4C). After 60 minutes there was no carboxyfluorescein signal detected on the boundaries or in the interior of the GUVs, showing that the addition of Tween 20 alone does not promote content-mixing between GUVs and LUVs. Additionally, CP_4_K_4_-Tween 20 GUVs were transferred to a microscope chamber and free carboxyfluorescein was added. The final concentration of carboxyfluorescein was adjusted to 1 µM in the microscope chamber, which gives similar background fluorescence to that detected in the membrane fusion experiments after the arrival of LUVs in the bottom of the chamber. Quantification of fluorescence inside individual GUVs showed that there was no carboxyfluorescein present in the interior of the GUVs 60 minutes after the experiment was started, (Fig. 4C, lower panel) confirming that Tween 20 does not alter the membrane permeability of the GUVs such that they become destabilized or leaky. In addition, several control experiments were performed to validate coiled-coil driven membrane fusion in the presence of Tween 20 (Supplementary Figs. S8–S13). Removing CP_4_K_4_ from the LUVs produced minimal clustering and no content transfer from LUVs to non-functionalized GUVs. However, the appearance of fluorescent spots onto the target membrane of CP_4_K_4_-Tween 20 functionalized GUVs was visible upon the addition of non-functionalized LUVs, in agreement with the lipid-mixing experiments. Visual content mixing was detected in some GUVs (*i.e*. GUV 10 in Fig. S11) and confirmed with the development of fluorescence over time in some GUVs (Supplementary Figs. S10 and S11) indicative of non-specific content exchange.

Together, time-lapse lipid- and content-mixing experiments showed successful fusion of LUVs and GUVs. A strong interaction of CP_n_K_4_ with the lipid bilayer may cause destabilization of the GUV lipid membrane; however this can be regulated by changing the length of the PEG spacer. The incorporation of CP_n_K_4_ resulted in inhomogeneous fluorescence on the target membrane of individual GUVs due to LUV clustering, which makes the evaluation of content-mixing efficiency challenging. This effect can be modulated by the incorporation of Tween 20 with the lipopeptide CP_n_K_4_. The mixture of CP_n_K_4_-Tween 20 leads to an increase of content mixing events in a single GUV. Therefore, the effect of Tween 20 on the incorporation of CP_n_K_4_ and the effect of this mixture on the mobility of the membrane lipids was studied by z-scan fluorescence correlation spectroscopy (z-scan FCS)^57^.

### FRET experiments confirm both peptides are needed for efficient fusion to occur

**Figure 5:**
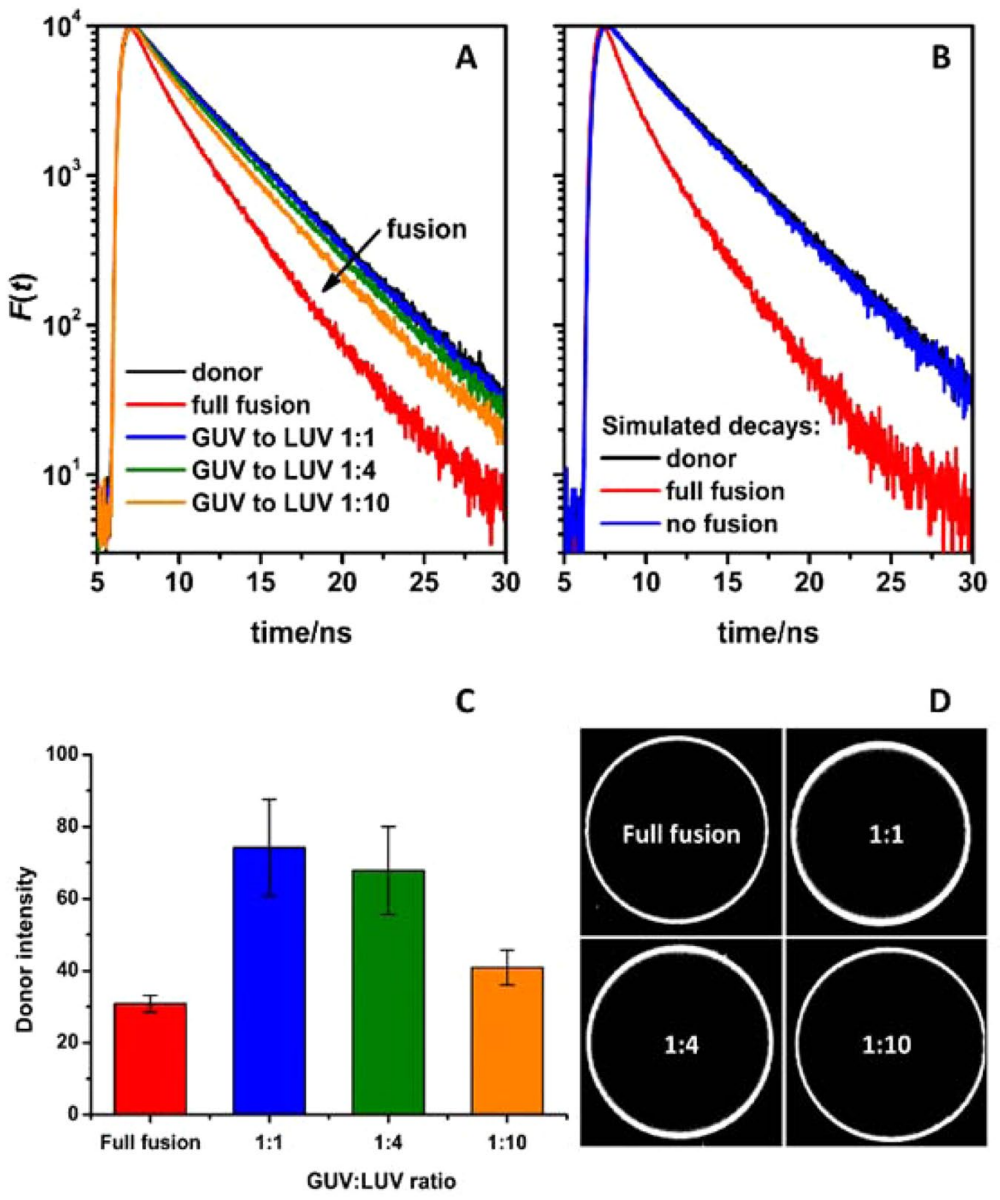
Fusion of CP_4_K_4_-containing GUVs with CP_4_E_4_-containing LUVs as monitored by time-resolved FRET. The GUVs decorated with 0.5 mol% of ATTO 488 DOPE (donor) were mixed with LUVs decorated with 0.5 mol% of ATTO 633 DOPE (acceptor) at various GUV to LUV concentrations. The full fusion conditions correspond to a hypothetical scenario where all LUVs fused with GUVs at the GUV:LUV ratio 1:1. The simulated decays shown in panel B correspond to the following scenarios: the decay of donors in the absence of acceptors (black); nonfusion conditions where the entire surface of the GUVs was saturated by attached LUVs (blue); and full fusion conditions (red). The corresponding intensities of the donors are shown in panel C. Representative images of GUVs are shown on panel D. The emission of the donors was recorded at 515 nm.

To confirm that the bilayers of the GUVs and LUVs do indeed fuse efficiently, time-resolved FRET experiments were employed. To perform FRET, ATTO 488 DOPE donors and ATTO 633 DOPE acceptors were incorporated at different GUV to LUV ratios (Fig. 5A). On one hand, simulated decays were calculated to ensure that in this experimental setup a detectable FRET develops only when the approaching bilayers fuse (see the Supplementary Section “FRET as a tool to monitor membrane fusion” online). From the simulated time-resolved fluorescence decays (Fig. 5, panel B), even under conditions when the GUV surface is fully saturated by LUVs, FRET is not strong enough to significantly affect the shape of the calculated decays. In contrast, fluorescent relaxation becomes significantly faster when full fusion occurs (compare the blue and red decay lines in Fig. 5, panel B). In FRET experiments, on the other hand, at lower GUV to LUV ratios (1:1 and 1:4), the detected FRET efficiency was clearly different from the non-fusion scenario in which the entire surface of the GUV would be saturated by LUVs (Fig. 5, panel A). The highest FRET was detected at a GUV:LUV ratio of 1:10, revealing that to obtain full lipid mixing, the GUV:LUV ratio would need to be even higher. In conclusion, these findings confirm that both CP_n_E_4_ and CP_n_K_4_ are necessary for efficient lipid-mixing and the visualization of membrane fusion requires high quantities of LUVs, due to the large difference in size between LUVs and GUVs.

### FCS experiments reveal Tween 20 does indeed soften lipid membranes

**Figure 6:**
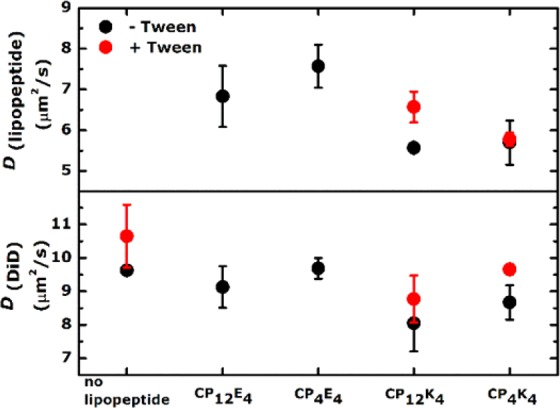
Diffusion coefficients (*D*) of CP_n_E_4_ and CP_n_K_4_ lipopeptides in GUVs in the absence (black dots) or presence (red dots) of Tween 20. The concentration of Tween 20 was 1 mol% of the total amount of the lipopeptide. Every point plotted corresponds to the average of at least three different GUVs and the error bars correspond to the standard deviation.

The diffusion coefficients (*D*) of the lipopeptides CP_n_E_4_ and CP_n_K_4_ (Fig. 6, upper panel) were compared to those of DiD (Fig. 6, lower panel), as DiD reflects the overall mobility of the lipid bilayer, which is sensitive to the presence of the lipopeptide incorporated. In general, diffusion coefficients of all lipopeptides were lower than *D*(DiD). This indicates that cholesterol strongly anchors the lipopeptides in the membranes of GUVs, with the cholesterol being well incorporated^58^. The *D*(DiD) tracer values in the absence of CP_n_E_4_ are similar to the values of the *D*(DiD) in the presence of lipopeptide CP_n_E_4_ regardless the length of PEG spacer, indicating similar membrane mobility. In contrast, a higher mobility restriction was found for *D*(DiD) in the presence of lipopeptide CP_n_K_4_, with a 10% decrease of the diffusion coefficient for the shorter PEG_4_ spacer and a 15% decrease with the longer PEG_12_ spacer in comparison with *D*(DiD) in the absence of CP_n_K_4_. This result confirms the strong K_4_–membrane interaction that was observed in the time-lapse lipid-mixing assay between CP_4_K_4_-functionalized GUVs and non-functionalized LUVs (Supplementary Fig. S1). The addition of Tween 20 increases the *D* of lipopeptide CP_n_K_4_ (n = 4, 12) for the longer PEG_12_ spacer, while the shorter PEG_4_ spacer remains the same. Interestingly, the *D*(DiD) increases in the presence of Tween 20 for all CP_n_K_4_ (n = 4, 12) combinations. This increase in the *D*(DiD) suggests the softening of the lipid membrane by Tween 20, which was corroborated in the absence of lipopeptides (Fig. 6, red dots in the lower panel). Together, these data indicate that the incorporation of Tween 20 facilitates membrane fusion by softening the lipid bilayer. Therefore, the CP_12_K_4_-Tween 20 mixture could be an optimal candidate for improving membrane fusion efficiency, but surfactant-lipopeptide interactions should be considered more deeply in further studies.

## Conclusions

We have successfully visualized coiled-coil driven membrane fusion between GUVs and LUVs at physiologically relevant ionic strengths with CaCl_2_ and MgCl_2_ supplemented PBS. The use of GUVs as a target membrane allowed the visualization of both lipid- and content-mixing assays by dual fluorescence color conventional microscopy. Time lapse imaging provided additional insights as to the mechanism of the membrane fusion process and facilitated optimization of the fusion model. Clustering of CP_n_K_4_-functionalized LUVs was detected in the membrane of GUVs, decreasing the efficiency of coiled-coil formation and hence fusion. Time-resolved FRET indicates that fusion occurs and is detectable only at high LUV:GUV ratios. Incorporation of the surfactant Tween 20 together with CP_n_K_4_ resulted in softening of the target membrane, leading to an improvement in the efficiency of fusion. These results indicate that this coiled-coil-based membrane fusion system could be applied as a fast and efficient cellular drug delivery system in future studies.

## Materials and Methods

### Materials

Details of all chemicals, synthesis of lipopeptides^28^, and formation of GUVs^46^, can be found in the Supplementary Information.

### Membrane fusion model system

GUVs with the lipid composition DOPC:DOPE:CH (2:1:1 molar ratio) were prepared by hydration of hybrid lipid/DexPEG hydrogel film substrates^46^. The lipid mixture was supplemented with ATTO 488 DOPE for fluorescence imaging during lipid-mixing experiments. The use of DexPEG substrates allows for the growth of GUVs under physiological conditions in good yields^59^. The lipid film was deposited on DexPEG substrates and hydrated at room temperature with phosphate buffer solution (PBS, pH = 7.4), containing CaCl_2_ (1 mM), MgCl_2_ (0.5 mM) and sucrose (200 mM). After GUV formation, the vesicles were transferred to a solution containing supplemented PBS and 1 mol% of CP_n_E_4_ or CP_n_K_4_ (n = 4, 12). The lipopeptides spontaneously insert into the GUV membrane via the cholesterol anchor (Fig. 1), which simulates the function of the transmembrane domain of SNARE proteins. Finally, peptide-functionalized GUVs were transferred to a microscopy chamber and were immobilized on the glass surface via streptavidin-biotin binding. The integrity of the GUVs was verified by fluorescence and bright field microscopy. In parallel, peptide-functionalized LUVs were prepared by sonication with the same lipid composition as used for GUVs, but with an ATTO 633 DOPE dye. The use of two different dyes for GUVs and LUVs avoids overlapping fluorescence signals in the lipid-mixing assays.

### Lipid mixing assays

LUV-GUV lipid mixing was initiated by treating CP_4_K_4_-functionalized GUVs (*circa* 20 µm diameter) with 30 µL CP_4_E_4_-functionalized LUVs (1 mM, *circa* 120 nm diameter) as represented in Fig. 1. LUVs were added to a microscope chamber containing 300 µL of immobilized GUVs in supplemented PBS. Instead of performing fluorescence resonance energy transfer (FRET) assays with NBD-rhodamine pairs, a technique commonly employed in bulk liposomal measurements^6,28,60,61^, we took advantage of the microscopic size of the GUVs and visualized the lipid bilayer of GUVs in a time-lapse dual-colour imaging microscopy experiment, which in the best of our knowledge, represents the first example of lipid mixing between LUVs and GUVs in the presence of complementary synthetic fusogens. The fluorescence signal from the GUVs was monitored at 500–550 nm and from the LUVs at 650–700 nm in a Leica TCS SPE confocal microscope every minute for one hour with LUV docking on GUV membranes being detected after a 20–35 minutes (Fig. 2).

### Content mixing assays

GUVs were prepared as described for lipid-mixing experiments, except that ATTO 633 DOPE was used in place of ATTO 488 DOPE. A solution of carboxyfluorescein at a self-quenching concentration (50 mM, pH 7) was encapsulated within the LUVs, and these were subsequently incubated with the appropriate lipopeptide (Fig. 1, right). In contrast to lipid mixing experiments, LUVs were formed by extrusion to increase the encapsulation efficiency of carboxyfluorescein. As the LUV solution was diluted by a factor of two after size exclusion, 60 µL of liposomes were added to the microscopy chamber in order to maintain a constant LUV concentration for both lipid- and content-mixing assays. GUVs were imaged for 60 minutes by dual fluorescence imaging (Fig. 3). The fluorescence signal was simultaneously monitored for both GUVs (633 nm laser, filter detection 650–700 nm) and LUVs (488 nm laser, filter detection 500–550 nm). A detailed analysis of each experiment is presented in the Supplementary Information Figs. S2–S14.

To probe content mixing further, an additional batch content-mixing experiment was performed in a microcentrifuge tube using CP_4_K_4_-functionalized GUVs and CP_4_E_4_-functionalized LUVs in the presence and absence of the nonionic surfactant Tween 20. After 60 minutes, the GUVs were transferred from the tube to a microscope chamber containing fresh, supplemented PBS solution for dual fluorescence imaging. This transfer process helped with the removal of background fluorescence, which is not possible to avoid during time lapse imaging (Fig. 4). The micrograph for a single CP_4_K_4_-functionalized GUV after membrane fusion is presented in Fig. 4A, no content mixing is observed, and for a single CP_4_K_4_-Tween 20 functionalized GUV in Fig. 4B, where content mixing is observed.

### Time-resolved förster resonance energy transfer (FRET)

Time-resolved FRET was used to monitor lipid mixing between GUVs and LUVs. GUVs were functionalized with 1 mol% of CP_4_K_4_ and 0.5 mol% of ATTO 488 DOPE (donor). Additionally, LUVs were functionalized with 1 mol% CP_4_E_4_ and 0.5 mol% of ATTO 633 DOPE (acceptor). Upon fusion of LUVs on the target membrane, the fluorescent donors come into proximity of ATTO 633 DOPE acceptors, which results in FRET. The increase of the FRET efficiency is manifested by a faster relaxation of the donors back to the ground state as presented in Fig. 5. The results of this experiment were compared with two limiting scenarios for which corresponding time-resolved fluorescence decays were generated (for details see the Supplementary Section “FRET as a tool to monitor membrane fusion” online): (a) a ‘no fusion’ scenario in which LUVs land on top of GUVs without fusing (blue decay in Fig. 5, panel B) and (b) a ‘full fusion’ scenario in which all LUVs fully fuse with GUVs (red decay in Fig. 5, panel B).

### Fluorescence correlation spectroscopy (FCS)

Z-scan FCS experiments were performed with fluorescent lipopeptides CP_n_K_4_-Atto 488 and CP_n_E_4_-Atto 488 (n = 4, 12). To facilitate this, a cysteine residue was introduced at the C-terminus of both peptides and the fluorescent dye ATTO 488 maleimide was coupled to this thiol-containing residue. The fluorescent lipopeptide to lipid ratio was set at 1:20,000, producing a final concentration of 0.005 mol% lipopeptide in the lipid membrane of GUVs. The experimental concentration of 1 mol% lipopeptide, which is used in lipid- and content-mixing assays was reached by mixing fluorescent lipopeptides with non-fluorescent lipopeptides at a ratio of 1:200. The lipid probe 1,1′-dioctadecyl-3,3,3′,3′-tetramethylindo-dicarbocyanine perchlorate (DiD) was used as a membrane tracer in a 1:100,000 dye:lipid ratio. The time-dependent intensity fluctuations were measured using z-scan FCS in order to calculate the autocorrelation function. This autocorrelation function was then fitted by a model which assumes free 2D diffusion, yielding lipopeptide and DiD diffusion coefficients (*D*) (Fig. 6).

### Labeling of GUV with lipopeptides CP_n_K_4_ and CP_n_E_4_

GUVs were functionalized with 1 mol% CP_n_K_4_ or CP_n_E_4_ (n = 4, 12). Stock solutions of CP_n_K_4_ or CP_n_E_4_ (28 µL, 50 µM in CH_3_OH:CHCl_3_ 1:1) were dried by evaporating the solvent under a gentle stream of air and subsequently placing in a vacuum oven overnight. The lipopeptide film was hydrated by adding 700 µL of PBS supplemented with CaCl_2_ (1 mM), MgCl_2_ (0.5 mM) and glucose (200 mM); this is referred to as supplemented PBS in this manuscript. The resulting lipopeptide solution was vortexed and transferred to a microcentrifuge tube. 300 µL of a solution of free-floating GUVs was transferred into the microcentrifuge tube containing the lipopeptide solution. The mixture was incubated for 60 minutes before 300 µL of the GUV-lipopeptide mixture was transferred to a microscopy chamber.

### Labeling of GUVs with lipopeptide CP_n_K_4_ and Tween 20

GUVs were functionalized with a mixture of 1 mol% CP_n_K_4_ and either 0.4 or 1 mol% (with respect to CP_n_K_4_) Tween-20. Stock solutions of CP_n_K_4_ (28 µL, 50 µM in CH_3_OH:CHCl_3_ 1:1) and Tween 20 (6 μL or 14 μL, 0.001 mM in CH_3_OH) were mixed and dried by evaporating the solvent under a stream of air before being placed in a vacuum oven overnight. The CP_n_K_4_-Tween 20 film was hydrated by adding 700 µL of supplemented PBS, vortexed and transferred to a microcentrifuge tube. Subsequently 300 µL of a solution of free-floating GUVs was transferred into the microcentrifuge tube containing the CP_4_K_4_-Tween 20 mixture. The solution was incubated for 60 minutes before 300 µL was transferred to a microscopy chamber.

### Formation of LUVs with lipopeptides CP_n_K_4_ or CP_n_E_4_ for lipid-mixing experiments

Peptide-functionalized LUVs were formed using 1 mol% CP_n_K_4_ or CP_n_E_4_ (n = 4, 12). Lipid solutions (1 mL) with the lipid composition 50 mol% 1,2-dioleoyl-sn-glycero-3-phosphocholine (DOPC), 25 mol% 1,2-dioleoyl-sn-glycero-3-phosphoethanolamine (DOPE) and 25 mol% cholesterol (CH) (2:1:1 molar ratio, 1 mM), a fluorescent lipid analogue ATTO 633 DOPE (0.5 mol%) and lipopeptides CP_n_K_4_ or CP_n_E_4_ (50 µM in CH_3_OH:CHCl_3_ 1:1) were mixed and dried by evaporating the solvent and placing in a vacuum oven overnight. The dried lipid film was rehydrated by adding 1 mL of PBS supplemented with CaCl_2_ (1 mM), MgCl_2_ (0.5 mM) and sucrose (200 mM). The LUVs were subsequently formed by sonication at room temperature for 2–4 minutes to form LUVs with ~120 nm diameters, as determined by DLS (Zetasizer Nano-S, Malvern).

### Formation of carboxyfluorescein-loaded LUVs with lipopeptides CP_n_K_4_ or CP_n_E_4_ for content mixing experiments

A lipid solution (1 mL) with the lipid composition DOPC:DOPE:CH (2:1:1 molar ratio, 1 mM) and 0.5 mol% ATTO 633 DOPE was prepared and subsequently dried under a gentle stream of air before being placed in a vacuum oven overnight. The lipid film was hydrated by adding 1 mL of carboxyfluorescein (50 mM) in PBS supplemented with CaCl_2_ (1 mM), MgCl_2_ (0.5 mM) and sucrose (200 mM). The LUVs were formed by extrusion (0.4 µm polycarbonate membrane) in a mini extruder fitted with 250 µL syringes. Free carboxyfluorescein was separated from the liposome-encapsulated carboxyfluorescein by size exclusion using a Sephadex column (2.5 mL) with supplemented PBS as the eluent. Liposome formation was verified by DLS. Finally, these LUVs were functionalized with 1 mol% CP_n_K_4_ or CP_n_E_4_. A stock solution of CP_n_K_4_ or CP_n_E_4_ (200 µL, 50 µM in CH_3_OH:CHCl_3_ 1:1) was dried under a gentle stream of air and placed in a vacuum oven overnight. The lipopeptide film was hydrated by adding 200 µL of supplemented PBS and was subsequently mixed with the LUV solution (*circa* 2.5 mL) for 60 minutes before being used immediately for content mixing experiments with GUVs.

### Imaging of GUVs during membrane fusion assays and data analysis

Imaging of GUVs was performed on a Leica TCS SPE confocal microscope system using 1-minute time between frames. Illumination was provided by a solid-state laser using a 488 nm laser (15% laser power) for irradiation of carboxyfluorescein and ATTO 488 DOPE, detection 500–550 nm, and a 635 nm laser (15% laser power) for irradiation of ATTO 633 DOPE, detection 650–700 nm. Analysis of the images was performed in ImageJ^62^, by measuring the average intensity of an area corresponding to one GUV for the series of time-lapsed microscopy image frames.

### Z-scan fluorescent correlation spectroscopy (z-scan FCS)

FCS measurements were performed on an inverted home-built confocal microscope (IX71 Olympus, Hamburg, Germany). Excitation was achieved by two pulsed diode lasers at 470 nm (LDH-P-C-470) and 635 nm (LDH-D-C-635) produced by PicoQuant, Germany. The laser light (10 µW intensity in front of the objective) was pulsed alternately in order to avoid artefacts caused by signal bleed-through. The emitted light was detected by two single-photon avalanche diodes using 515/50 and 697/58 band pass filters (Chroma Rockingham, VT). Z-scan measurements were performed on top of selected GUV. The membrane was vertically scanned in 15 steps spaced 200 nm apart. A measurement at each point took 60 s. All data was analyzed using home-written scripts in Matlab (Mathworks, Natick, MA).

## Supplementary information

Supporting information.
