# Supplementary material for: Controlled Peptide-Mediated Vesicle Fusion Assessed by Simultaneous Dual-Colour Time-Lapsed Fluorescence Microscopy
Source: Sci Rep. 2020 Feb 20;10:3087. doi: 10.1038/s41598-020-59926-z (PMC7033240; doi:10.1038/s41598-020-59926-z)
Supplement: Supplementary file 1 — Supporting information. [file 41598_2020_59926_MOESM1_ESM.docx]

Controlled Peptide-Mediated Vesicle Fusion Assessed by Simultaneous Dual-Colour Time-Lapsed Fluorescence Microscopy

*Nestor Lopez Mora,^1^ Aimee L. Boyle,^1^ Bart Jan van Kolck,^1^ Anouk Rossen,^1^ Šárka Pokorná,^2^ Alena Koukalová,^2^ Radek Šachl,^2^ Martin Hof ^2*^ and Alexander Kros.^1*^*

*^1^ Supramolecular and Biomaterials Chemistry, Leiden Institute of Chemistry, Leiden University, P.O. Box 9502, 2300 RA Leiden, The Netherlands.*

*^2^ J. Heyrovský Institute of Physical Chemistry, Academy of Sciences of the Czech Republic, v.v.i., Dolejškova 2155/3, 182 23 Prague 8, Czech Republic.*

SUPPLEMENTARY INFORMATION

**Contents**: Page

1. **EXPERIMENTAL SECTION**

Chemicals SI 4

Synthesis of lipopeptides CP_4_K_4_ and CP_4_E_4_. SI 4

Synthesis of lipopeptides CP_12_K_4_ and CP_12_E_4_. SI 5

Fluorescent lipopeptides CP_n_K_4_ and CP_n_E_4_. SI 5

Formation of GUVs. SI 5

Sample preparation and imaging for lipid and content mixing assays. SI 6

Sample preparation for z-scan FCS. SI 6

Description of supporting video of the content mixing experiment with the mixture GUV CP_12_K_4_-Tween 20 – LUV CP_12_E_4_ blue and green channels. SI 7

1. **DATASETS FOR TIME LAPSE LIPID AND CONTENT-MIXING AND CONTROL EXPERIMENTS BETWEEN GUV AND LUV.**

Supplementary Figure S1. Time lapse lipid-mixing control experiments between GUVs and LUVs. SI 8

Supplementary Figure S2-3. Content-mixing between CP_4_K_4_-GUVs and CP_4_E_4_-LUVs loaded with carboxyfluorescein. SI 9

Supplementary Figure S4-5. Content mixing between CP_4_K_4_-GUVs (Tween20) 0.4 mol% and CP_4_E_4_-LUVs loaded with carboxyfluorescein. SI 10

Supplementary Figure S6-7. Content mixing between CP_4_K_4_-GUVs (Tween20) 1 mol% and CP_4_E_4_-LUVs loaded with carboxyfluorescein. SI 11

Supplementary Figure S8-9. Control experiment between GUVs (Tween20) 1 mol% and LUVs loaded with carboxyfluorescein. SI 12

Supplementary Figure S10-11. Control experiment between CP_4_K_4_-GUVs (Tween20) 1 mol% and LUVs loaded with carboxyfluorescein. SI 13

Supplementary Figure S12-13. Control experiment between GUVs (Tween20) 1 mol% and CP_4_E_4_-LUVs loaded with carboxyfluorescein. SI 14

Supplementary Figure S14. Leakage of 1 µM carboxyfluorescein into CP_4_K_4_-GUVs (Tween20) 1 mol%. SI 15

1. **FRET AS A TOOL TO MONITOR MEMBRANE FUSION** SI 16
2. **REFERENCES** SI 18
3. **EXPERIMENTAL SECTION**

***Chemicals.***

1,2-dioleoyl-sn-glycero-3-phosphocholine (DOPC), 1,2-dioleoyl-sn-glycero-3-phosphoethanolamine (DOPE), 1,2-dioleoyl-sn-glycero-3-phosphoethanolamine-N-(biotinyl) (sodium salt) (DOPE-Biotin), were purchased from Avanti Polar Lipids. Rink Amide ChemMatrix resin, Cholesterol (CH), Bovine Serum Albumin (BSA), biotin-labeled bovine albumin (Biotin-BSA), Streptavidin from *Streptomyces avidinii*, polyethylene glycol sorbitan monolaurate (Tween 20), anhydrous calcium chloride (CaCl_2_), magnesium chloride hexahydrate (MgCl_2_·6 H_2_O), 5(6)-carboxyfluorescein, sodium hydroxide (NaOH), sucrose and glucose were purchased from Sigma-Aldrich. 1,2-dioleoyl-sn-glycero-3-phosphoethanolamine-ATTO 488 (ATTO 488 DOPE), 1,2-dioleoyl-sn-glycero-3-phosphoethanolamine-ATTO 633 (ATTO 633 DOPE), ATTO 488 maleimide and ATTO 655 maleimide were purchased from ATTO-TEC GmbH. 1,1′-dioctadecyl-3,3,3′,3′-tetramethylindo-dicarbocyanine perchlorate (DiD) was supplied by Life Technologies Corporation (Carlsbad, CA). Fmoc-protected amino acids were purchased from Novabiochem and Biosolve. All solvents were purchased from Biosolve or Honeywell. Phosphate Buffered Saline (PBS, pH 7.4) was supplemented with CaCl_2_ (1 mM) and MgCl_2_ (0.5 mM) for all GUV studies. Lipid solutions of DOPC:DOPE:CH (50:25:25 molar ratio, 1 and 14 mM) were prepared in chloroform.

***Synthesis of lipopeptides CP_4_K_4_ and CP_4_E_4_.***

The spacer N_3_-PEG_4_-COOH, cholesteryl- 4-amino-4-oxobutanoic acid, and the lipopeptides CP_4_K_4_ and CP_4_E_4_ were synthesized and utilized following procedures previously reported.[^1-3^](#_ENREF_1) The peptide segments E: NH_2_-(EIAALEK)_4_-CONH_2_ and K: NH_2_-(KIAALKE)_4_-CONH_2_ were synthesized using standard Fmoc chemistry on a peptide synthesizer (CEM-Liberty 1), then the spacer N_3_-PEG_4_-COOH was coupled to the N-terminus of the peptide segment with 3 equivalents of HCTU and 4 equivalents of DIPEA. The azide terminal group on the spacer was reduced to an amine to obtain an N-terminal free amine for coupling to cholesteryl-4-amino-4-oxobutanoic acid using 5 eq. of DIPEA and 4 eq. of PyBOP in DMF over 72 h. Finally, the lipopeptides were purified by RP-HPLC with a Gemini C4 column to yield a pure product. The identity of the peptides and lipopeptides was determined by LC-MS.

***Synthesis of lipopeptides CP_12_K_4_ and CP_12_E_4_.***

The peptide segments E: NH_2_-(EIAALEK)_4_-GC-CONH_2_ and K: NH_2_-(KIAALKE)_4_-GC-CONH_2_ were synthesized using standard Fmoc chemistry on a peptide synthesizer (CEM-Liberty 1). After synthesis, the resin was washed with DMF and Fmoc-PEG_12_-COOH (1.1 equivalents) was coupled to the N-terminus of the peptides using 3 equivalents of HCTU and 4 equivalents of DIPEA. The reaction was left to proceed overnight. The resin was then washed with DMF, before the PEG_12_ molecule was Fmoc-deprotected using a 20% piperidine in DMF solution. Deprotection was achieved by incubating the resin with the piperidine/DMF solution for 10 minutes. This process was repeated 3 times, after which the resin was washed with DMF. Cholesteryl hemisuccinate was coupled using the same methodology as for the PEG coupling, (3 equivalents of HCTU and 4 equivalents of DIPEA, overnight reaction). The resin was washed with DMF, followed by DCM, before the product was cleaved from the resin using a mixture of TFA:TIPS:EDT (95:2.5:2.5). The cleavage solution was left for one hour before the peptide was precipitated in ice-cold diethyl ether. The peptide was collected by centrifugation before being dissolved in water and freeze dried. Finally, the lipopeptides were purified by RP-HPLC with a Gemini C4 column to yield a pure product. The identity of the peptides and lipopeptides was determined by LC-MS.

***Fluorescent lipopeptides CP_n_K_4_ and CP_n_E_4_.***

Lipopeptides were fluorescently labeled with ATTO 488 and ATTO 655 dyes via a maleimide-thiol reaction. Lipopeptides modified with a cysteine residue at the N-terminus (1 mg) were dissolved in 1 mL DCM and a 1.3 fold molar excess of ATTO maleimide dye solution (1 mg/mL) was added. The reaction mixture was stirred for 1 hour, whilst being protected from light. The solvent was evaporated and the lipopeptides were purified by RP-HPLC with a Gemini C4 column. The identity of the fluorescently labeled lipopeptides was determined by MALDI-TOF Mass Spectrometry.

***Formation of GUVs.***

Giant Unilamellar Vesicles (GUVs) were grown on Dex-PEG hydrogel (1:1 molar ratio) coated microscope glass slide substrates as described previously.[^4^](#_ENREF_4)^,^[^5^](#_ENREF_5) Lipid solution (10 µL) with the lipid composition DOPC/DOPE/CH (2/1/1 molar ratio, 14 mM), DOPE-Biotin (0.2 mol % respect to lipids) and ATTO 488 DOPE for lipid mixing experiments or ATTO 633 DOPE for content mixing experiments (0.1 mol % respect to lipids) was deposited on a hydrogel-coated glass slide, then the lipid solution was dried by evaporating the chloroform under a gentle stream of air and subsequently placed in a vacuum oven overnight. A liquid chamber was made by placing a 15 mm (OD) glass O-Ring on top of the hydrogel and sealed with high vacuum silicon grease. The lipid film was hydrated by adding 400 μL of PBS supplemented with CaCl_2_ (1 mM), MgCl_2_ (0.5 mM) and sucrose (200 mM) into each chamber and the GUVs were formed overnight at room temperature.

***Sample preparation and imaging for lipid and content mixing assays.***

The visualization of GUVs after lipopeptide labeling was achieved using a microscopy chamber which was pre-treated with an aqueous mixture of BSA (0.9 mg/mL) and biotin-BSA (0.1 mg/mL) for 30 minutes, followed by streptavidin for 30 minutes before rinsing with water. 100 µL of supplemented PBS and 200 µL of peptide-functionalized GUVs were transferred into the microscopy visualization chamber. The GUVs were left to sediment for 30 minutes before imaging. During imaging of the GUVs in a time lapse experiment, either 30 µL of peptide-functionalized LUVs, for a lipid-mixing experiment, or 60 µL of peptide-functionalized LUVs loaded with carboxyfluorescein for content-mixing experiments were added to the microscopy well and imaging was performed for 120 minutes after the arrival of LUVs into the microscopy chamber.

***Sample preparation for z-scan FCS.***

Giant unilamellar vesicles (GUVs) were prepared by electroformation,[^6^](#_ENREF_6)^,^[^7^](#_ENREF_7) with the lipid composition DOPC:DOPE:Cholesterol (2:1:1). Additionally, the lipid mixture was supplemented with DiD in a lipid ratio 1:100,000 as membrane tracer and biotinylated DOPE (2 mol%) for immobilization of GUVs. The lipid mixture was spread on two hollow titanium electrode plates. Solvent evaporation was facilitated by mild heating of the plates and successive drying in vacuum for at least 1 hour. An electroformation chamber was made by putting together two plates, filled with 275 mOsm sucrose solution and sealed with parafilm. Electroformation was performed following the successive voltage (V) sequence at 40 °C: 1) V (peak to peak) increased from 0.02 to 1.1 V at 10 Hz for 45 minutes, 2) V = 1.1 V at 10 Hz for 100 minutes and 3) V = 1.3 V at 4 Hz for 30 minutes. An observation chamber (Nunc® Lab-Tek® Chamber) was coated with biotin-BSA followed by streptavidin and filled with 360 μl phosphate buffer (25mM PBS, 100mM KCl, 1mM EDTA, ~ 20 mM glucose, pH 7.4, 275 mOsm). 40 μl of the GUV sucrose solution was incubated for 30 minutes with CP_n_K_4_ or CP_n_E_4_ and transferred to the observation chamber. For the experiments with Tween 20 the lipopeptides were mixed with 1 mol% Tween 20 (with respect to the concentration of CP_n_K_4_). For FCS diffusion measurements lipopeptides fluorescently labelled with Atto-488 were used in a 1:20,000 lipopeptide:lipid ratio. Higher concentrations of the lipopeptides were achieved by mixing fluorescent labelled lipopeptides with unlabeled ones. GUVs were left for at least 20 minutes and immobilized via a biotinylated DOPE-streptavidin-biotin-BSA linker to the glass on the bottom of the chamber. Measurements were carried out at room temperature.

***Description of supporting video of the content mixing experiment with the mixture GUV CP_12_K_4_-Tween 20 – LUV CP_12_E_4_ blue and green channels.***

CP_12_K_4_-Tween 20 – LUV CP_12_E_4__blue channel. GUVs are excited at 633 nm and fluorescence emission is detected between 650-700 nm. The movie shows three fluorescently-labelled GUVs, two of them with a size *circa* 20 µm (left and right) and one with a size of *circa* 40 µm (center). The integrity of the GUV lipid bilayer can be followed during the time lapse content-mixing experiment. The large vesicle bursts at 0.02 seconds in the movie while the small vesicles remain until the end of the experiment without any alteration.

CP_12_K_4_-Tween 20 – LUV CP_12_E_4__green channel. GUVs are excited at 488 nm and the emission is detected between 500-550 nm. Imaging of the same GUV is described in blue channel. The movie shows the fluorescence of carboxyfluorescein encapsulated in the LUVs. At 0.01 seconds docking of LUVs can be seen in the 20 µm GUV (left) and 40 µm GUV (centre), while the 20 µm GUV on the right does not show visible docking. At 0.02 seconds the 40 µm GUV (centre) blasts and finally at 0.03 seconds content mixing can be observed in 20 µm GUV on the left, but no content mixing is observed in the 20 µm GUV on the right.

1. **DATASETS FOR TIME LAPSE LIPID-, AND CONTENT-MIXING AND CONTROL EXPERIMENTS BETWEEN GUVs AND LUVs.**

*Time lapse lipid mixing control experiments between GUVs and LUVs.*

**
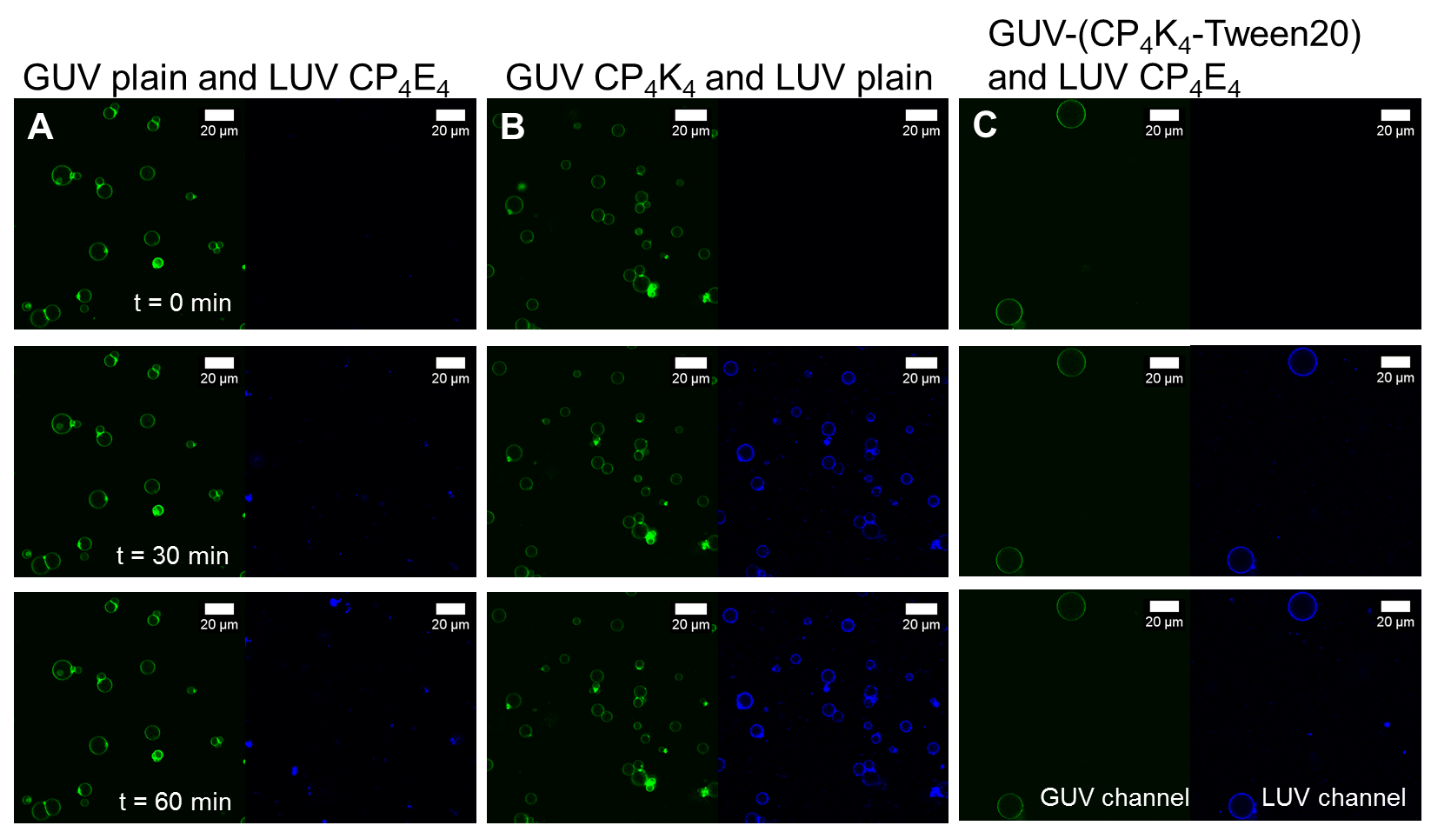
** **Supplementary Figure S1.** Time lapse micrographs of the lipid mixing control assay between GUVs and LUVs before (time=0) and after (time=30 and 60 minutes) appearance of LUVs in the confocal volume. The GUVs are excited at 488 nm and the emission of fluorescence is detected between 500-550 nm (green), while LUVs are excited at 633 nm and the emission is detected between 650-700 nm (blue). A) Lipid-mixing assay between GUVs and CP_4_E_4_-LUVs, B) lipid-mixing assay between CP_4_K_4_-GUVs and LUVs and C) lipid-mixing assay between CP_4_K_4_-GUVs (Tween20) and CP_4_E_4_-LUVs. Imaging was performed every minute for one hour in a Leica TCS SPE microscope.

*Content mixing between CP_4_K_4_-GUVs and CP_4_E_4­_-LUVs loaded with carboxyfluorescein.*

**
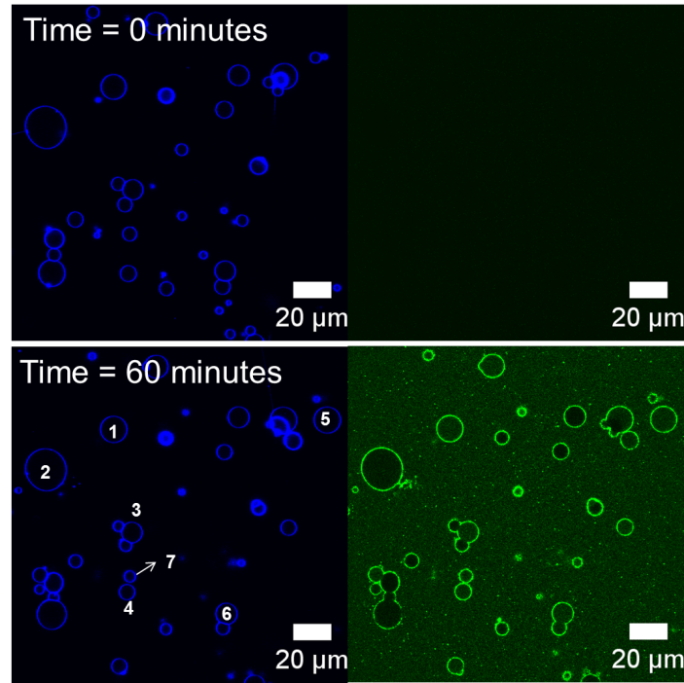
**


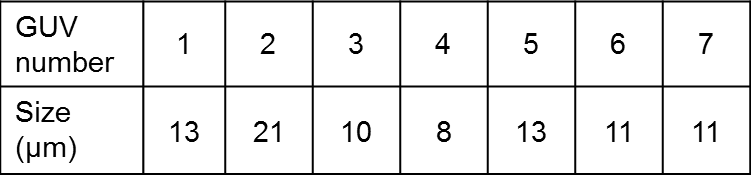


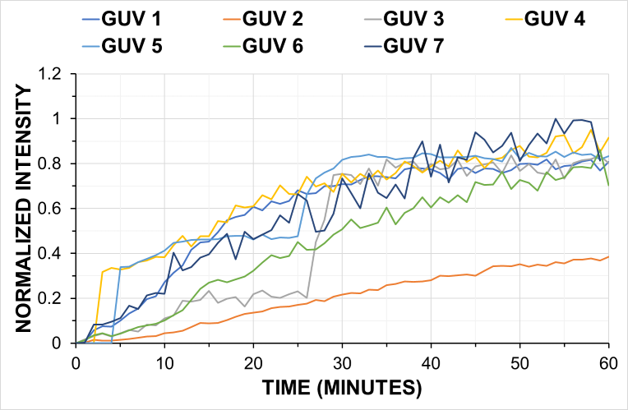


Supplementary Figure S2. Fluorescence micrographs before (top) and after (bottom) addition of carboxyfluorescein-loaded CP_4_E_4_-LUVs to CP_4_K_4_-GUVs. GUV lipid membranes are supplemented with ATTO 633 DOPE (blue) and LUVs are loaded with carboxyfluorescein (green). On the right, normalized fluorescence intensity profiles over time for individually numbered GUVs and the average background can be observed, and in the upper panel the average diameter over time for GUVs is shown in the table.

**
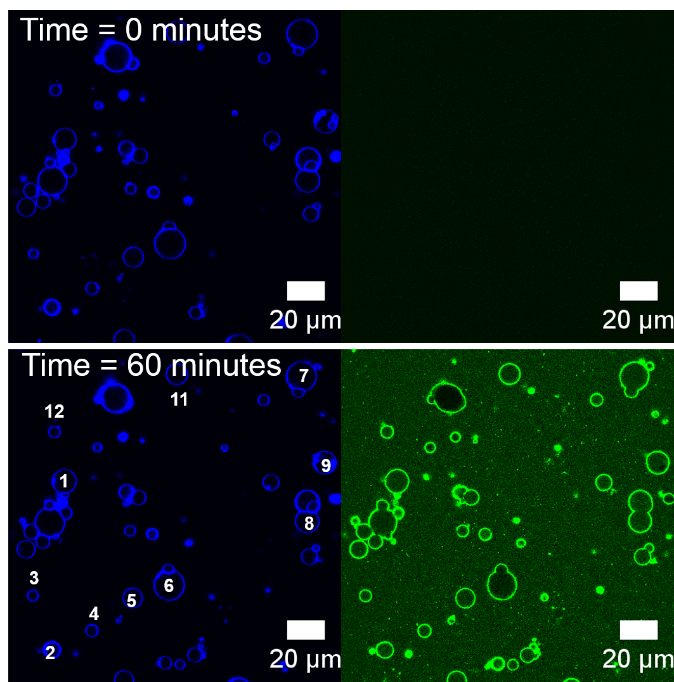
**


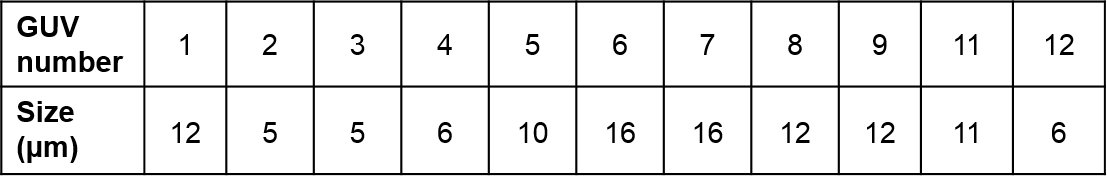


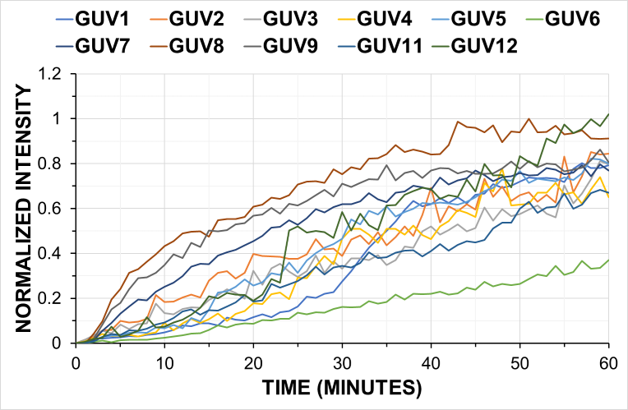


Supplementary Figure S3. Fluorescence micrographs before (top) and after (bottom) addition of carboxyfluorescein-loaded CP_4_E_4­_-LUVs to CP_4_K_4_-GUVs. GUV lipid membranes are supplemented with ATTO 633 DOPE (blue) and LUVs are loaded with carboxyfluorescein (green). On the right, normalized fluorescence intensity profiles over time for individually numbered GUVs and the average background are shown, and in the upper panel the average diameter over time for the GUVs is displayed in the table.

*Content mixing between CP_4_K_4_-GUVs (Tween20) 0.4 mol% and CP_4_E_4_-LUVs loaded with carboxyfluorescein.*

**
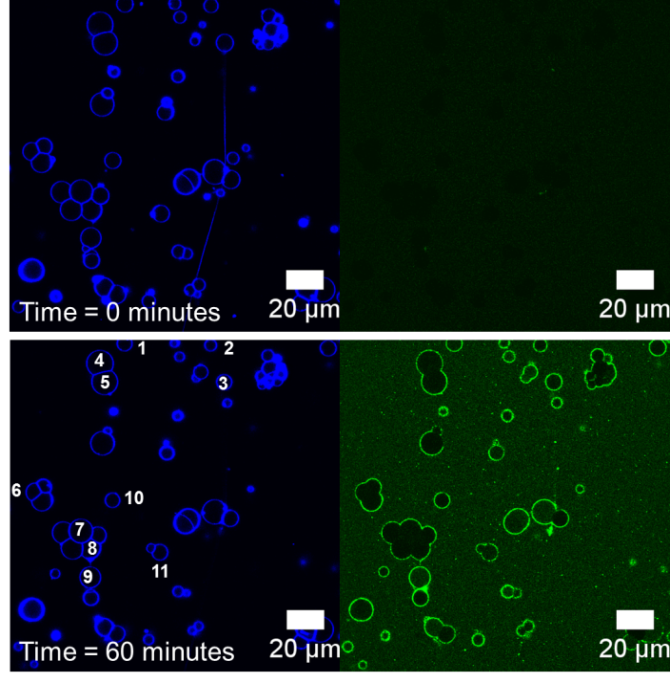
**


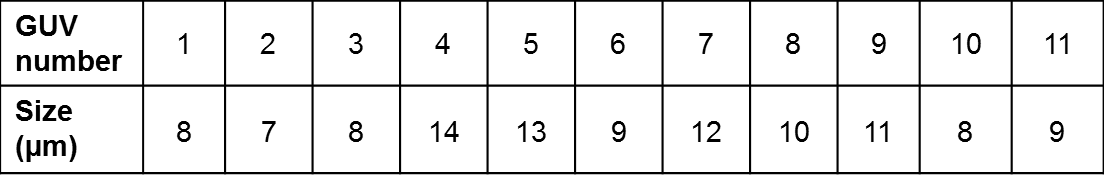


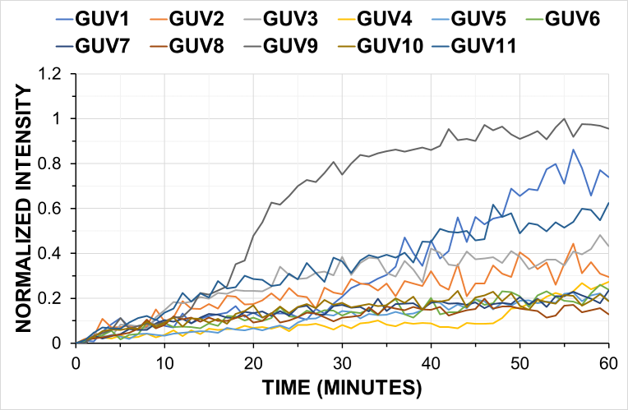


Supplementary Figure S4. Fluorescence micrographs before (top) and after (bottom) addition of carboxyfluorescein-loaded CP_4_E_4_-LUVs to CP_4_K_4_-GUVs (Tween20). GUV lipid membranes are supplemented with ATTO 633 DOPE (blue) and LUVs are loaded with carboxyfluorescein (green). On the right, normalized fluorescence intensity profiles over time for individually numbered GUVs and the average background fluorescence intensity are shown, and the table in the upper panel gives the average diameter over time for the GUVs.

**
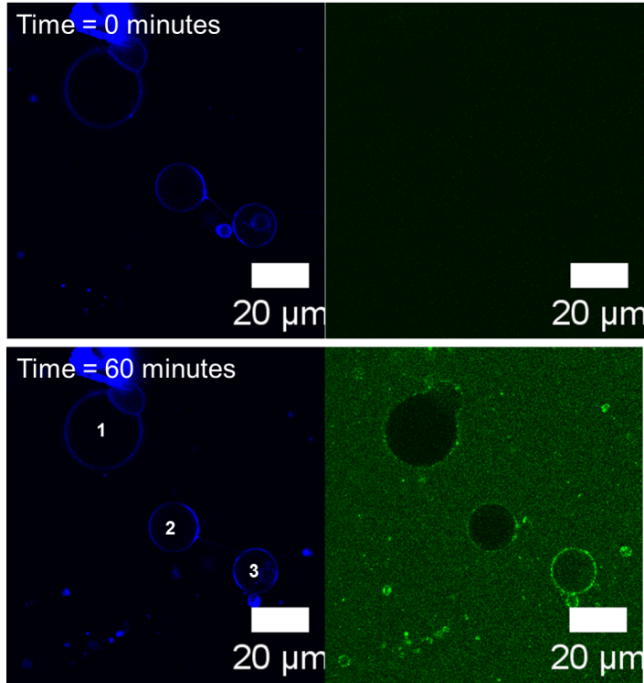
**


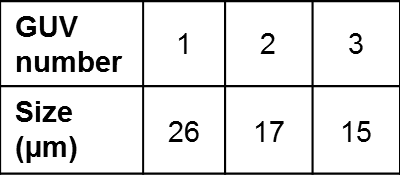


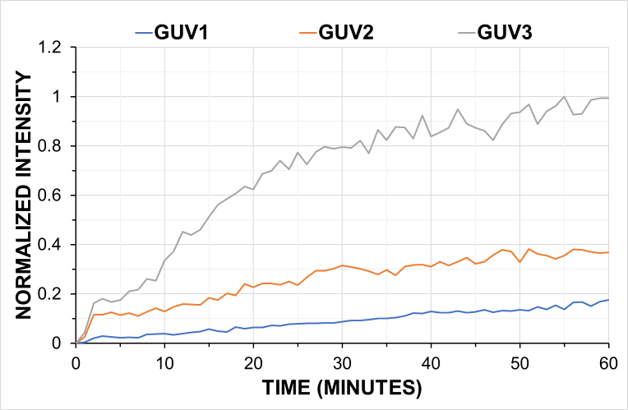


Supplementary Figure S5. Fluorescence micrographs before (top) and after (bottom) addition of carboxyfluorescein-loaded CP_4_E_4_-LUVs to CP_4_K_4_-GUVs (Tween20). GUV lipid membranes are supplemented with ATTO 633 DOPE (blue) and LUVs are loaded with carboxyfluorescein (green). On the right, normalized fluorescence intensity profiles over time for individually numbered GUVs and the average background intensity are shown, and in the upper panel the average diameter over time for GUVs is given in the table.

*Content mixing between CP_4_K_4_-GUVs (Tween20) 1 mol% and CP_4_E_4_-LUVs loaded with carboxyfluorescein.*

**
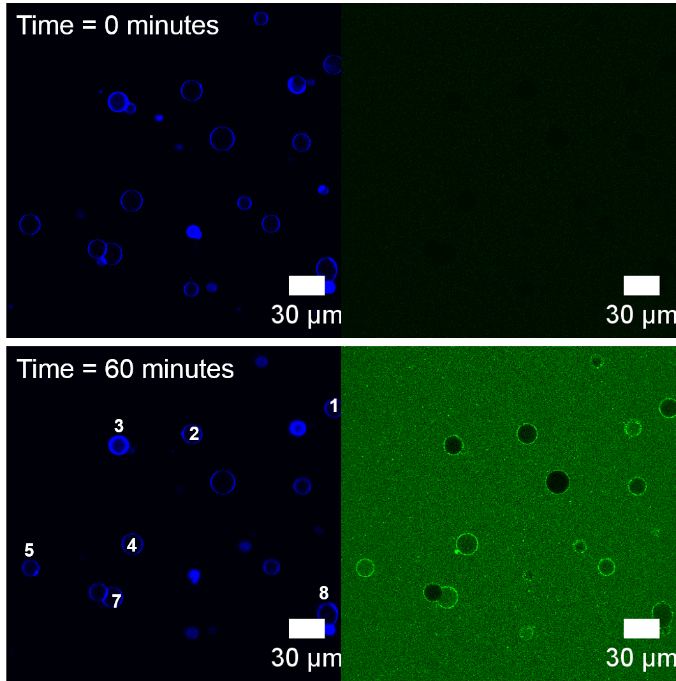
**


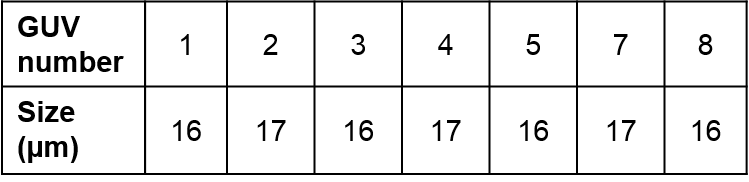


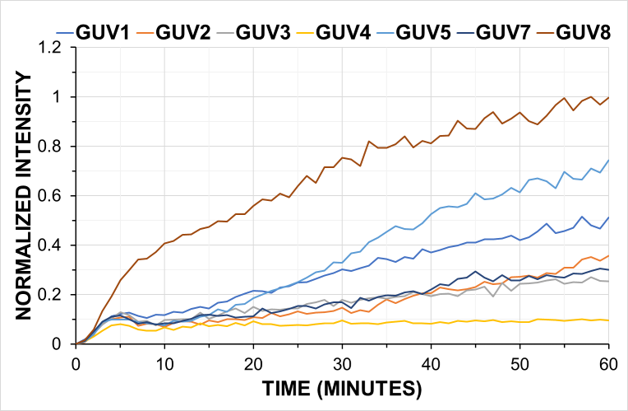


Supplementary Figure S6. Fluorescence micrographs before (top) and after (bottom) addition of carboxyfluorescein-loaded CP_4_E_4_-LUVs to CP_4_K_4_-GUVs (Tween20). GUV lipid membranes are supplemented with ATTO 633 DOPE (blue) and LUVs are loaded with carboxyfluorescein (green). On the right, normalized fluorescence intensity profiles over time for individually numbered GUVs and the average background are displayed, and in the upper panel the average diameter over time for the GUVs is shown.

**
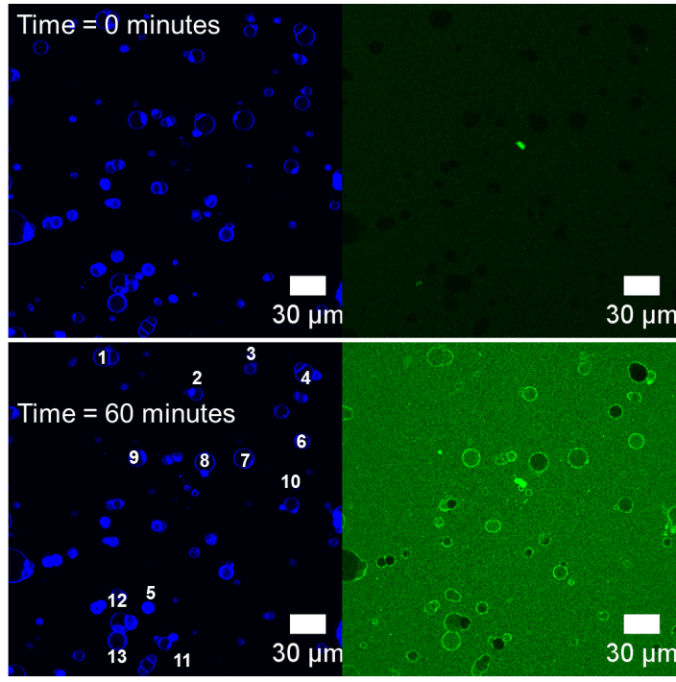
**


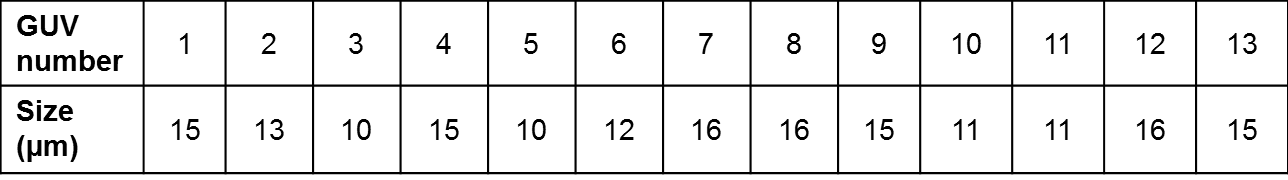


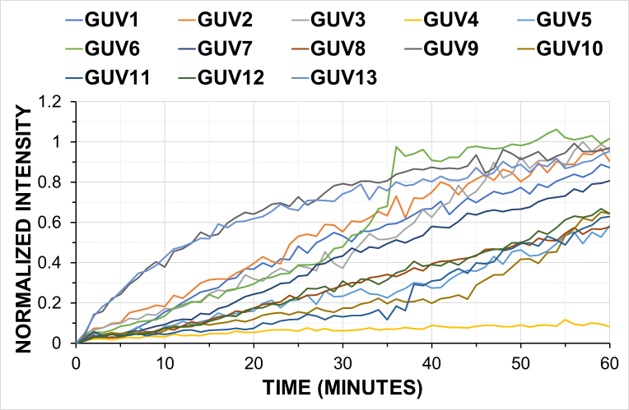


Supplementary Figure S7. Fluorescence micrographs before (top) and after (bottom) addition of carboxyfluorescein-loaded CP_4_E_4_-LUVs to CP_4_K_4_-GUVs (Tween20). GUV lipid membranes are supplemented with ATTO 633 DOPE (blue) and LUVs are loaded with carboxyfluorescein (green). On the right, normalized fluorescence intensity profiles over time are shown for individually numbered GUVs and for the average background and in the upper panel the table shows the average diameter over time for the GUVs.

*Control experiment between GUVs (Tween20) 1 mol% and LUVs loaded with carboxyfluorescein.*

**
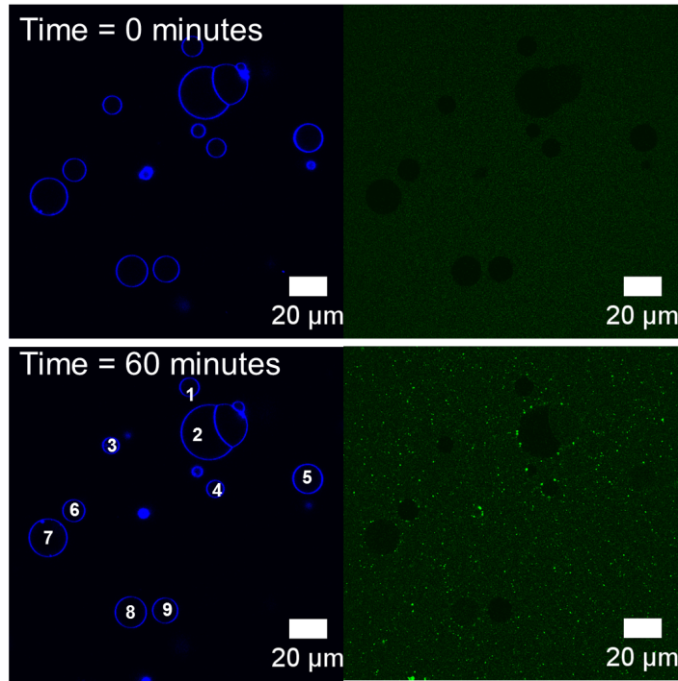
**


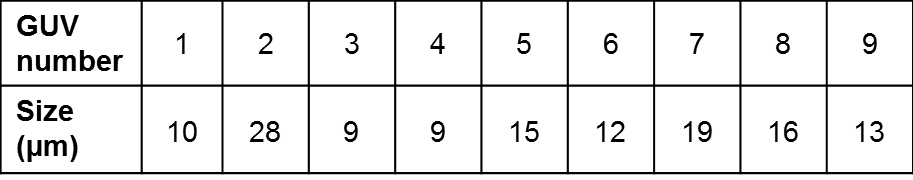


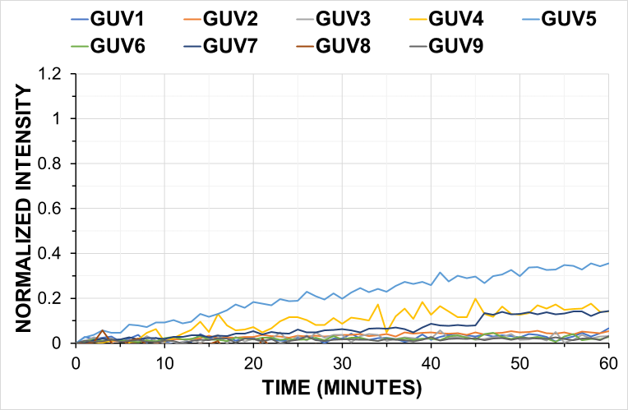


Supplementary Figure S8. Fluorescence micrographs before (top) and after (bottom) addition of carboxyfluorescein-loaded LUVs to GUVs (Tween20). GUV lipid membranes are supplemented with ATTO 633 DOPE (blue) and LUVs are loaded with carboxyfluorescein (green). On the right, normalized fluorescence intensity profiles over time for the individually numbered GUVs and the average background are shown and in the upper panel the average diameter over time for the GUVs is given.

**
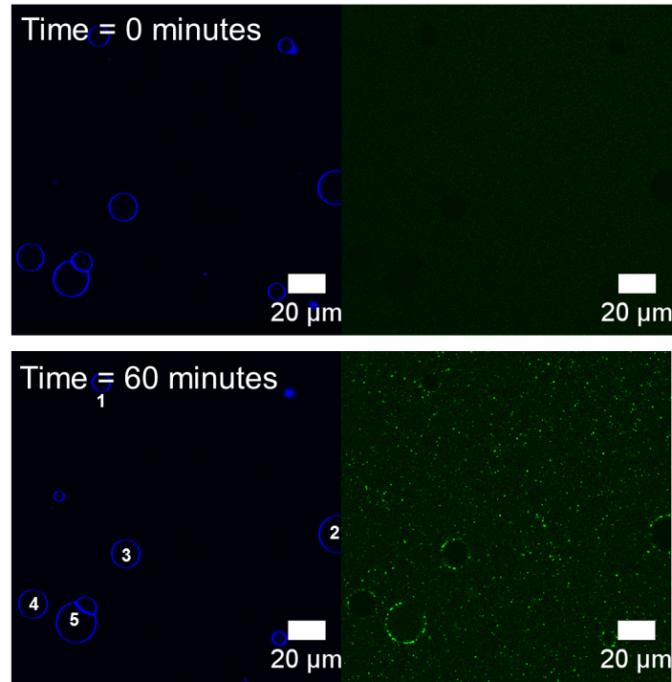
**


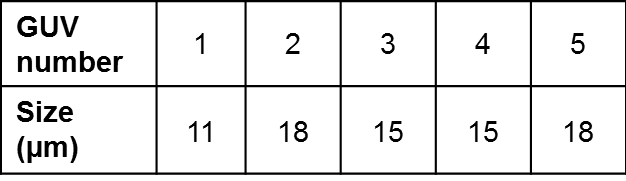


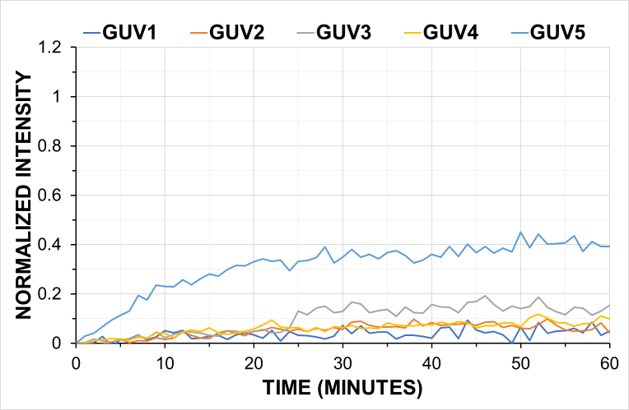


Supplementary Figure S9. Fluorescence micrographs before (top) and after (bottom) addition of carboxyfluorescein-loaded LUVs to GUVs (Tween20). GUV lipid membranes are supplemented with ATTO 633 DOPE (blue) and LUVs are loaded with carboxyfluorescein (green). On the right, normalized fluorescence intensity profiles over time for individually numbered GUVs and the average background are shown, and the table in the upper panel gives the average diameter over time for the GUVs.

*Control experiment between* *CP_4_K_4_-GUVs (Tween20) 1 mol% and LUVs loaded with carboxyfluorescein.*

**
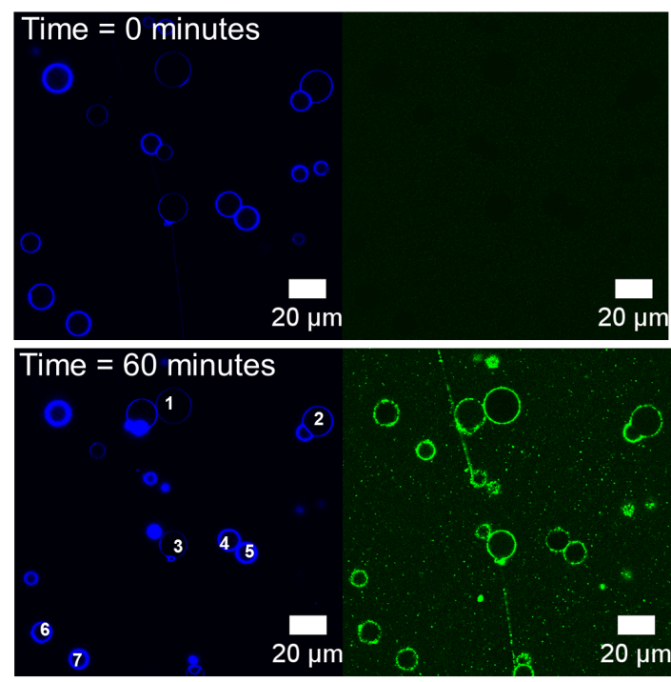
**


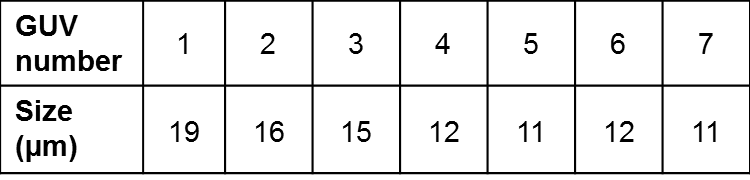


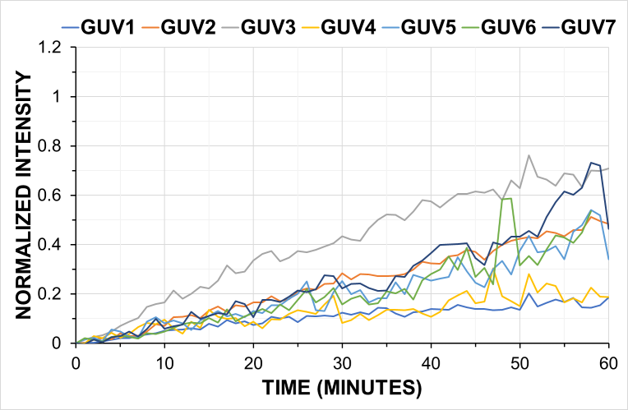


Supplementary Figure S10. Fluorescence micrographs before (top) and after (bottom) addition of carboxyfluorescein-loaded LUVs to CP_4_K_4_-GUVs (Tween20). GUV lipid membranes are supplemented with ATTO 633 DOPE (blue) and LUVs are loaded with carboxyfluorescein (green). On the right, normalized fluorescence intensity profiles over time for individually numbered GUVs and the average background fluorescence intensity are shown in the graph and in the upper panel the average diameter over time for the GUVs is given.

**
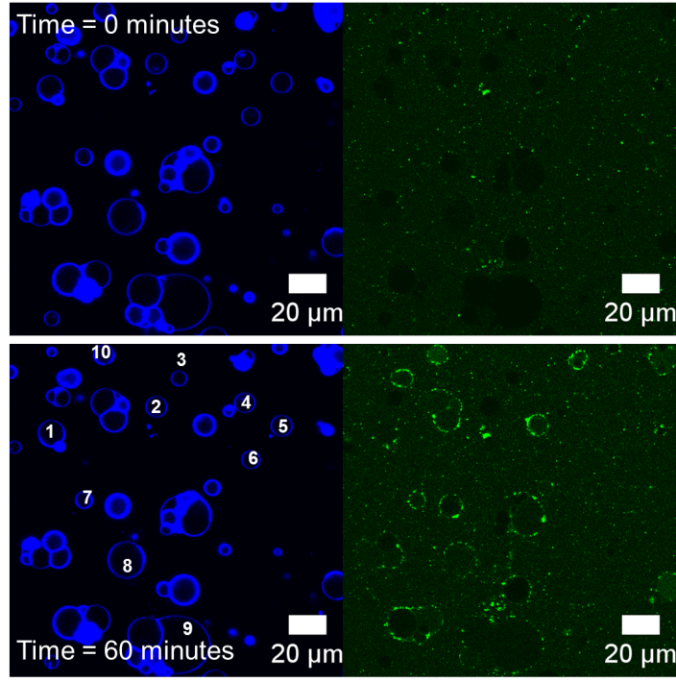
**


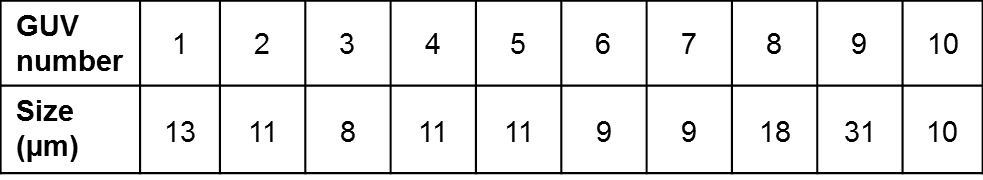


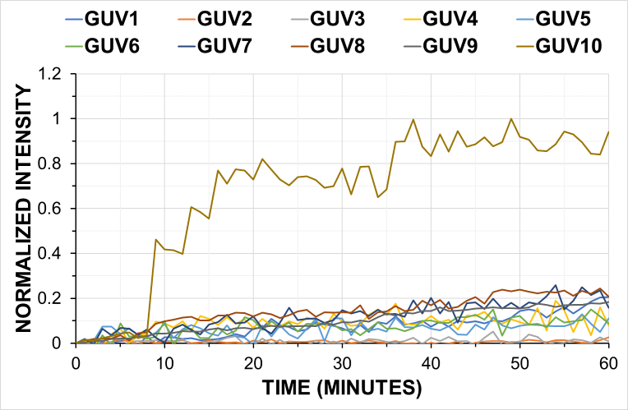


Supplementary Figure S11. Fluorescence micrographs before (top) and after (bottom) addition of carboxyfluorescein-loaded LUVs to CP_4_K_4_-GUVs (Tween20). GUV lipid membranes are supplemented with ATTO 633 DOPE (blue) and LUV are loaded with carboxyfluorescein (green). On the right, normalized fluorescence intensity profiles over time for individually numbered GUVs and the average background are displayed, and in the upper panel the average diameter over time for the GUVs is shown in the table.

*Control experiment between GUV (Tween20) 1 mol% and CP_4_E_4_-LUVs loaded with carboxyfluorescein.*

**
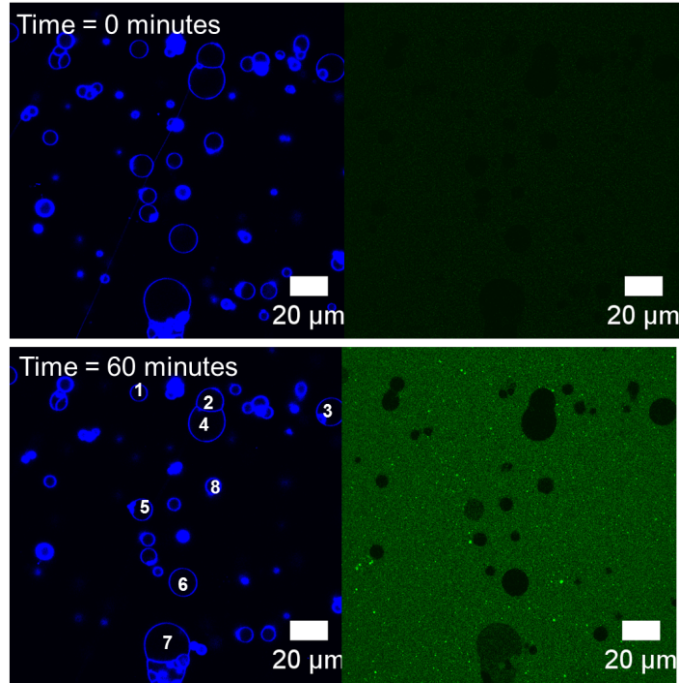
**


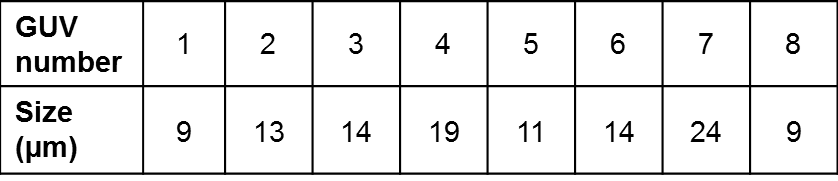


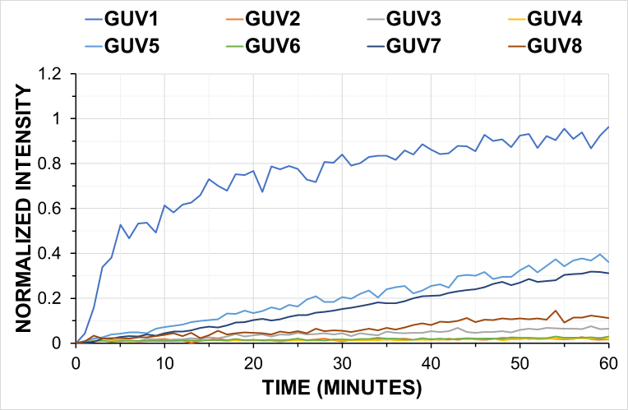


Supplementary Figure S12. Fluorescence micrographs before (top) and after (bottom) addition of carboxyfluorescein-loaded CP_4_E_4­_-LUVs to GUVs (Tween20). GUV lipid membranes are supplemented with ATTO 633 DOPE (blue) and LUVs are loaded with carboxyfluorescein (green). On the right, normalized fluorescence intensity profiles over time for individually numbered GUVs and the average background intensity is displayed and the table in the upper panel shows the average diameter over time for the GUVs.

**
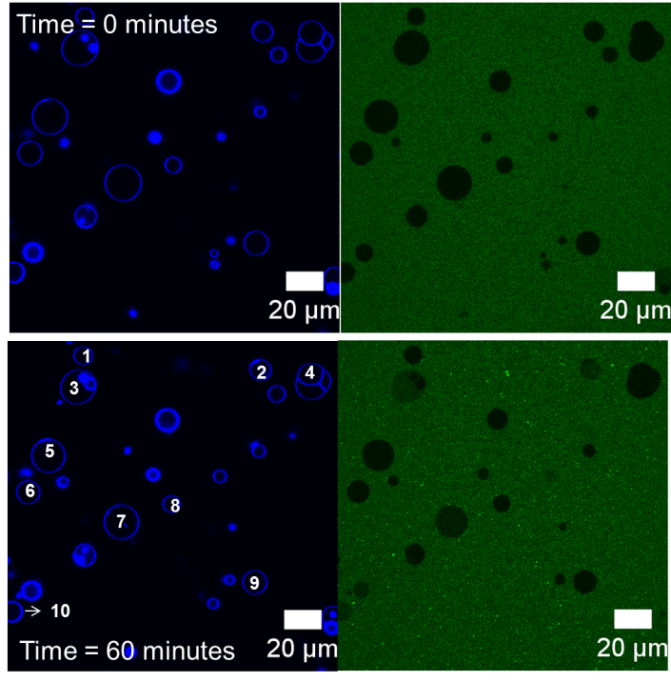
**


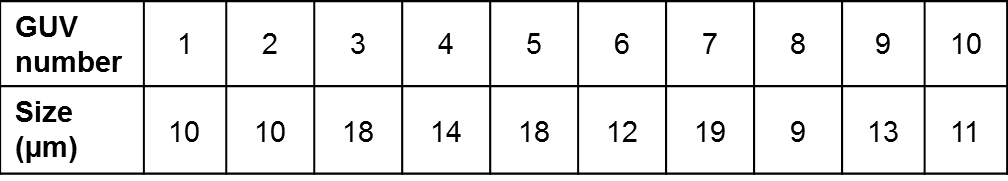


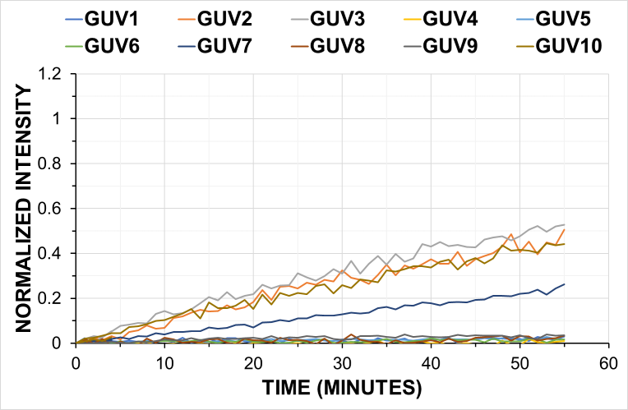


Supplementary Figure S13. Fluorescence micrographs before (top) and after (bottom) addition of carboxyfluorescein-loaded CP_4_E_4­_-LUVs to GUVs (Tween20). GUV lipid membranes are supplemented with ATTO 633 DOPE (blue) and LUVs are loaded with carboxyfluorescein (green). On the right, normalized fluorescence intensity profiles over time for individually numbered GUVs and the average background are shown, and in the upper panel the average diameter over time for the GUVs are given in the table.

*Leakage of 1 µM carboxyfluorescein into CP_4_K_4_-GUVs (Tween20) 1 mol%.*

**
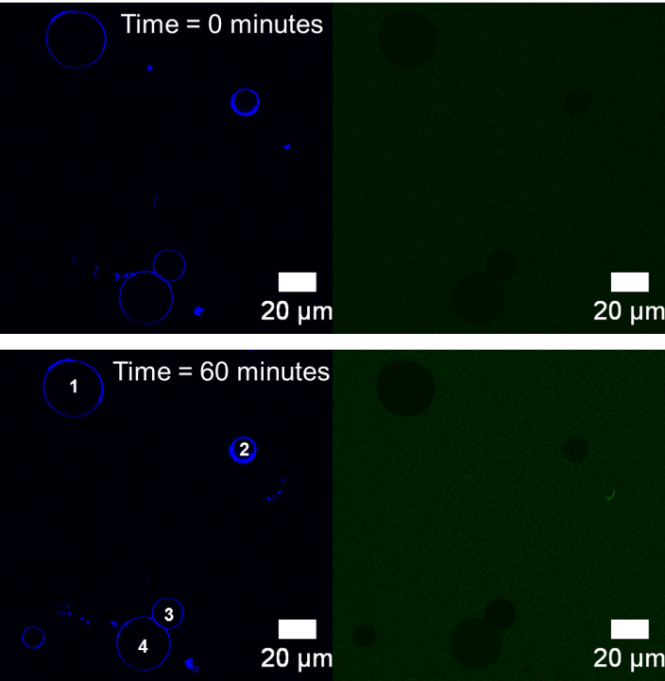
**


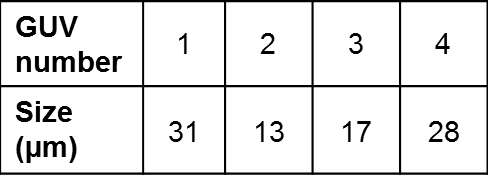


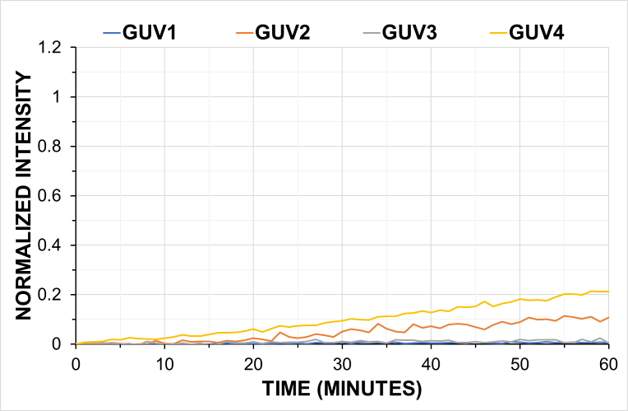


Supplementary Figure S14. Fluorescence micrographs before (top) and after (bottom) addition of 1 µM carboxyfluorescein to CP_4_K_4­_-GUVs (Tween20). GUV lipid membranes are supplemented with ATTO 633 DOPE (blue) and LUVs are loaded with carboxyfluorescein (green). On the right, normalized fluorescence intensity profiles over time for individually numbered GUVs and the average background are shown in the graph, and in the upper panel the average diameter over time for the GUVs are given in the table.

Note:

The normalized intensity is calculated following the equation:

$$Normalized intensity= \frac{I-I_{0}}{I_{max}- I_{0}}$$

Where *I_0_* is the intensity at time zero and *I_max_* is the highest intensity in the experiment.

1. **FRET AS A TOOL TO MONITOR MEMBRANE FUSION**

To monitor fusion between CP_4_K_4_ decorated GUVs and CP_4_E_4_ decorated LUVs, FRET (Förster resonance energy transfer) between ATTO 488 DOPE donors (dissolved in GUVs at 0.5 mol%) and ATTO 633 DOPE acceptors (dissolved in LUVs at 0.5 mol%) was used. This experiment is based on the assumption that FRET only arises if the approaching bilayers fuse together.

Initially, calculations verified that the scenario where LUVs land on the surface of GUVs does not lead to any detectable FRET. As shown in Supplementary Fig. S16, even in the extreme case when the surface of a GUV is fully covered by the LUVs, FRET can occur only within a very small region. The size of this region was estimated at 16% of the total surface area for vesicles that are 100 nm in diameter and are in direct contact with the surface of a GUV. FRET is not constant within this area: it is the highest at point B on Supplementary Fig. S16 and Supplementary Fig. S17 and decreases when moving towards point A and beyond. Since both bilayers are separated by the Förster radius *R*_0_ at point A, practically no FRET takes place beyond this point. In the calculations, an extreme case was simulated, and this was based on the following assumption: FRET occurs only within the ‘effective FRET area’ (Supplementary Fig. S16) where it does not change and is the same as in point B. These assumptions lead to overestimation of the FRET rate, but allow for generating a time-resolved fluorescence decay by modified equations of Baumann and Fayer (see Supplementary Box 1 and Figure 5 in main manuscript).

Specifically, simulated decay was generated by the following equation:

$F\left( t \right)=\left\{ f_{1}\left[ \frac{1}{2}G_{intra}\left( t \right)G_{inter}\left( t,d_{A} \right)+\frac{1}{2}G_{inter}\left( t,d_{A} \right)G_{inter}\left( t,d_{B} \right) \right]+\left( 1-f_{1} \right) \right\}F_{D}(t)$, (1)

where *F_D_*(*t*) is the decay of the donors in the absence of acceptors, *f*_1_ is the fraction of the surface of a GUV on which FRET takes place and (1- *f*_1_) is the fraction of the surface where donors cannot be quenched by the acceptors.

A decay for the case of full fusion was also generated (Figure 5, main manuscript)

$F\left( t \right)={G_{intra}\left( t \right)G_{inter}\left( t,d_{A} \right)F}_{D}(t)$. (2)

And finally, experimental decays were recorded (Figure 5) which were compared to the extreme cases that were simulated (Supplementary Equation 1 and Supplementary Equation 2).

| **Supplementary Box 1**  The so-called survival probability function for *intra*-FRET *G*_intra_ (i.e. FRET which occurs within the same leaflet, point B in Supplementary Fig. S17) is calculated as: (J. Baumann and M. D. Fayer, *J. Chem. Phys.*, 1986, **85**, 4087–4107)  $\ln G_{intra}\left( t \right)=-C_{2}\Gamma\left( \frac{2}{3} \right)\left( \frac{t}{\tau} \right)^{1/3}$ , (B1)  Here, *C_2_* is the reduced surface concentration of acceptors, which represents the average number of acceptors within the area of $\pi R_{0}^{2}$, *Γ* is the gamma function and *τ* the average lifetime of donors in the absence of acceptors. Similarly, the survival probability for *inter*-FRET, *G*_inter_, (i.e. FRET which occurs between two parallel leaflets, point B in Supplementary Fig. S17) is given by (J. Baumann and M. D. Fayer, *J. Chem. Phys.*, 1986, **85**, 4087–4107)  $\ln G_{inter}\left( t \right)=-\frac{C_{2}}{3}\left( \frac{d}{R_{0}} \right)^{2}\left( \frac{2\mu}{3} \right)^{1/3}\int_{0}^{2/{3\mu}} \left( 1-e^{-s} \right)s^{{-4}/3}ds$, (B2)  where *d* is the transversal distance between the layer of donors and the layer of acceptors, *θ_r_* is the angle between the bilayer normal and the vector connecting the locations of the donor and acceptor dipoles, $\mu=3t\left( \frac{R_{0}}{d} \right)^{6}\frac{1}{2\tau}$ and $s=2\mu\cos^{6} \frac{\theta_{r}}{3}$. If both *inter*- and *intra*-FRET occur simultaneously, the total survival probability is given by the joint probability $G\left( t \right)=G_{\text{intra}}\left( t \right)G_{\text{inter}}\left( t \right)$. The fluorescence intensity $F\left( t \right)$ of the donors in the presence of the acceptors is described as  $F\left( t \right)=G\left( t \right)\sum_{i} \alpha_{i}exp\left( -\frac{t}{\tau_{i}} \right)$, (B3)  where $\sum_{i} \alpha_{i}exp\left( {-t}/{\tau_{i}} \right)$ represents the decay of the donors in the absence of FRET. |
| --- |


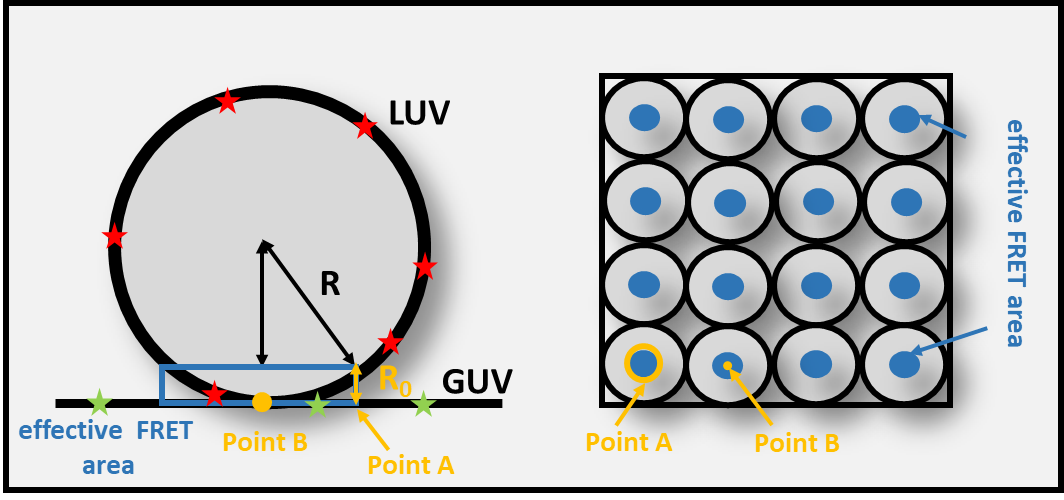


**Supplementary Figure S16:** (left) A side view of an LUV attached to the surface of a GUV. Point B indicates the place where the LUV is in direct contact with the GUV. At this place, FRET occurs as outlined by point B on Supplementary Fig. S17. Similarly, Point A indicates the place where the surfaces of a GUV and an LUV are found at the distance *R*_0_ from each other. At this place, FRET occurs as outlined by point A on Supplementary Fig. S17. Donors are marked by green stars, acceptors by the red ones. (right) A top view of the GUV surface which is fully crowded by attached LUVs. The effective FRET area denotes such a part of the GUV surface which is at maximum at the distance *R*_0_ from the surface of an LUV.


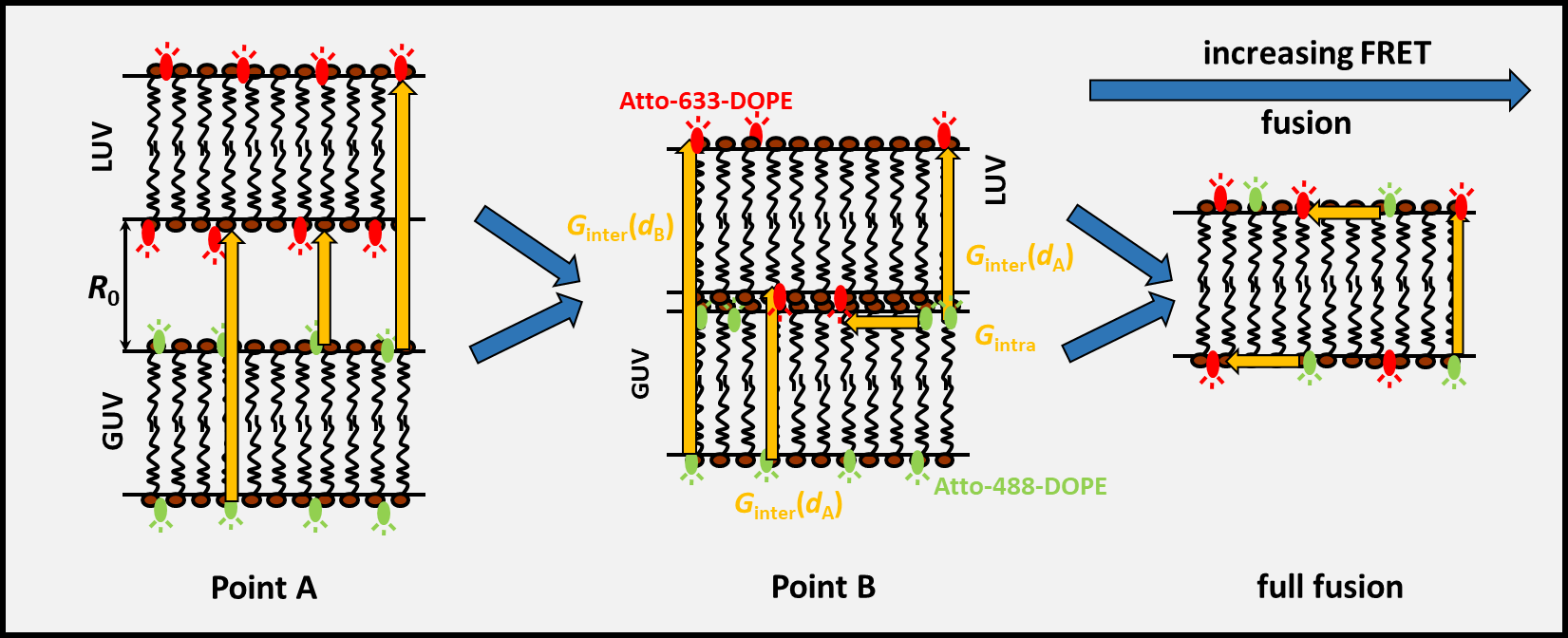


**Supplementary Figure S17.** A schematic representation of FRET which occurs between two lipid bilayers separated by the distance *R*_0_ (Point A), between two bilayers that are in direct contact (Point B) or on a fused lipid bilayer (full fusion). The donors (represented by ATTO 488 DOPE, green balls) were contained only in the membranes of GUVs whereas the acceptors (represented by ATTO 633 DOPE, red balls) were contained only in the membranes of LUVs.

1. **REFERENCES**

(1) Voskuhl, J.; Wendeln, C.; Versluis, F.; Fritz, E. C.; Roling, O.; Zope, H.; Schulz, C.; Rinnen, S.; Arlinghaus, H. F.; Ravoo, B. J.; Kros, A. *Angew Chem Int Edit* **2012**, *51*, 12616.

(2) Marsden, H. R.; Elbers, N. A.; Bomans, P. H. H.; Sommerdijk, N. A. J. M.; Kros, A. *Angew Chem Int Edit* **2009**, *48*, 2330.

(3) Versluis, F.; Voskuhl, J.; van Kolck, B.; Zope, H.; Bremmer, M.; Albregtse, T.; Kros, A. *Journal of the American Chemical Society* **2013**, *135*, 8057.

(4) Mora, N. L.; Hansen, J. S.; Gao, Y.; Ronald, A. A.; Kieltyka, R.; Malmstadt, N.; Kros, A. *Chem Commun* **2014**, *50*, 1953.

(5) Mora, N. L.; Gao, Y.; Gutierrez, M. G.; Peruzzi, J.; Bakker, I.; Peters, R.; Siewert, B.; Bonnet, S.; Kieltyka, R. E.; van Hest, J. C. M.; Malmstadt, N.; Kros, A. *Soft Matter* **2017**, *13*, 5580.

(6) Angelova, M. I.; Soleau, S.; Meleard, P.; Faucon, J. F.; Bothorel, P. *Prog Coll Pol Sci S* **1992**, *89*, 127.

(7) Koukalova, A.; Pokorna, S.; Fiser, R.; Kopecky, V., Jr.; Humpolickova, J.; Cerny, J.; Hof, M. *Biochimica et biophysica acta* **2015**, *1848*, 444.
